# Supplementary material for: Determining the effects of pseudouridine incorporation on human tRNAs
Source: EMBO J. 2025 Apr 29;44(13):3553–85. doi: 10.1038/s44318-025-00443-y (PMC12217144; doi:10.1038/s44318-025-00443-y)
Supplement: Supplementary file 1 — Appendix [file 44318_2025_443_MOESM1_ESM.pdf]

Appendix

Determining the effects of pseudouridine incorporation on human tRNAs

Authors: Anna D. Biela[1], Jakub S. Nowak[1], Artur P. Biela[1], Sunandan Mukherjee[2], Seyed Naeim Moafinejad[2], Satyabrata Maiti[2], Andrzej Chramiec-Głąbik[1], Rahul Mehta[1,3], Jakub Jeżowski[1], Dominika Dobosz[1], Priyanka Dahate[1], Veronique Arluison[4,5], Frank Wien[6], Paulina Indyka[1,7], Michal Rawski[1,7], Janusz M. Bujnicki[2,*], Ting-Yu Lin (林亭妤)[1,8,*] and Sebastian Glatt[1,9,*]

Affiliations:

[1] Malopolska Centre of Biotechnology, Jagiellonian University, 30-387, Krakow, Poland

[2] Laboratory of Bioinformatics and Protein Engineering, International Institute of Molecular and Cell Biology in Warsaw, 02-109, Warsaw, Poland

[3] Doctoral School of Exact and Natural Sciences, Jagiellonian University, 30-348, Krakow, Poland

[4] Laboratoire Léon Brillouin LLB, UMR12 CEA CNRS, CEA Saclay, 91191, Gif-sur-Yvette, France

[5] Université Paris Cité, UFR Sciences du vivant, 75006, Paris cedex, France

[6] Synchrotron SOLEIL, L'Orme des Merisiers, Saint Aubin BP48, 91192, Gif-sur-Yvette, France

[7] National Synchrotron Radiation Centre SOLARIS, Jagiellonian University, 30-392, Krakow, Poland

[8] Department of Biosciences, Durham University, DH1 3LE, Durham, UK

[9] University of Veterinary Medicine Vienna, 1210 Vienna, Austria

*Correspondence: janusz@iimcb.gov.pl (J.M.B.), ting-yu.lin@durham.ac.uk (T-Y.L.) and sebastian.glatt@uj.edu.pl (S.G.; @GlattSebastian)

Table of contents

Appendix Figure S1 **Page 3**

Sequences and predicted 2D structures of tRNAs used in this study.

Appendix Figure S2 **Page 4**

Cryo-EM reconstruction pipeline for unmodified $tRNA^{Gly}_{CCC}$.

Appendix Figure S3 **Page 5**

Cryo-EM reconstruction pipeline for PUS 4/7 modified $tRNA^{Gln}_{UUG}$.

Appendix Figure S4 **Page 6**

Cryo-EM reconstruction pipeline for PUS3/7 modified $tRNA^{Gln}_{UUG}$.

Appendix Figure S5 **Page 7**

Cryo-EM reconstruction pipeline for PUS4/7 modified $tRNA^{Gly}_{CCC}$.

Appendix Figure S6 **Page 8**

Cryo-EM reconstruction pipeline for unmodified $tRNA^{Glu}_{UUC}$.

Appendix Figure S7 **Page 9**

Cryo-EM reconstruction pipeline for PUS4/7 modified $tRNA^{Glu}_{UUC}$.

Appendix Figure S8 **Page 10**

Cryo-EM reconstruction pipeline for unmodified $tRNA^{Asp}_{GUC}$.

Appendix Figure S9 **Page 11**

Cryo-EM reconstruction pipeline for PUS4/7 modified $tRNA^{Asp}_{GUC}$.

Appendix Figure S10 **Page 12**

Detection of PUS-dependent Ψ formation on $tRNA^{His}_{GUG}$, $tRNA^{Lys}_{UUU}$, $tRNA^{Ser}_{UGA}$, $tRNA^{Arg}_{UCU}$.

Appendix Figure S11 **Page 13**

Cryo-EM reconstruction pipeline for PUS3/4/7 modified $tRNA^{Gly}_{CCC}$.

Appendix Figure S12 **Page 14**

Local resolution in regions around the respective modification sites of tRNAs.

A

tRNA	Sequence
Arg_TCT_4-1	GUCUCUGUGGCGCAAUGGACGAGCGCGCUGGACUUCUAAUCCAGAGGUUCCGGGUUCGAGUCCCGGCAGAGAUG
Asp_GTC_2-1	**GG**UCCUCGUUAGUAUAGUGGUGAGUAUCCCCGCCUGUCACGCGGGAGACCGGGGUUCGAUUCCCCGACGGGGAGCCA
Gln_TTG_3-1	GGCCCCAUGGUGUAAUGGUUAGCACUCUGGACUUUGAAUCCAGCGAUCCGAGUUCAAAUCUCGGUGGGACCUCCA
Glu_TTC_4-1	**GG**UCCCUGGUGGUCUAGUGGCUAGGAUUCGGCGCUUUCACCGCCGCGGCCCGGGUUCGAUUCCCGGUCAGGGAACCA
Gly_CCC_2-1	GCGCCGCUGGUGUAGUGGUAUCAUGCAAGAUUCCCAUUCUUGCGACCCGGGUUCGAUUCCCGGGCGGCGCACCA
His_GTG_1-1	GCCGUGAUCGUAUAGUGGUUAGUACUCUGCGUUGUGGCCGCAGCAACCUCGGUUCGAAUCCGAGTCACGGCACCA
Lys_TTT_3-1	GCCCGGAUAGCUCAGUCGGUAGAGCAUCAGACUUUUAAUCUGAGGGUCCAGGGUUCAAGUCCCUGUUCGGGCGCCA
Pro_TGG_3-1	GGCUCGUUGGUCUAGGGGUAUGAUUCUCGCUUUGGGUGCGAGAGGUCCCGGGUUCAAAUCCCGGACGAGCCCCA
Ser_TGA_1-1	**G**GCAGCGAUGGCCGAGUGGUUAAGGCGUUGGACUUGAAAUCCAAUGGGGUCUCCCCGCGCAGGUUCGAACCCUGCUCGCUGCGCCA
Val_TAC_4-1	**G**GUUUCCGUGGUGUAGUGGUUAUCACAUUCGCCUUACACGCGAAAGGUCCUCGGGUCGAAACCGAGCGGAAACACCA

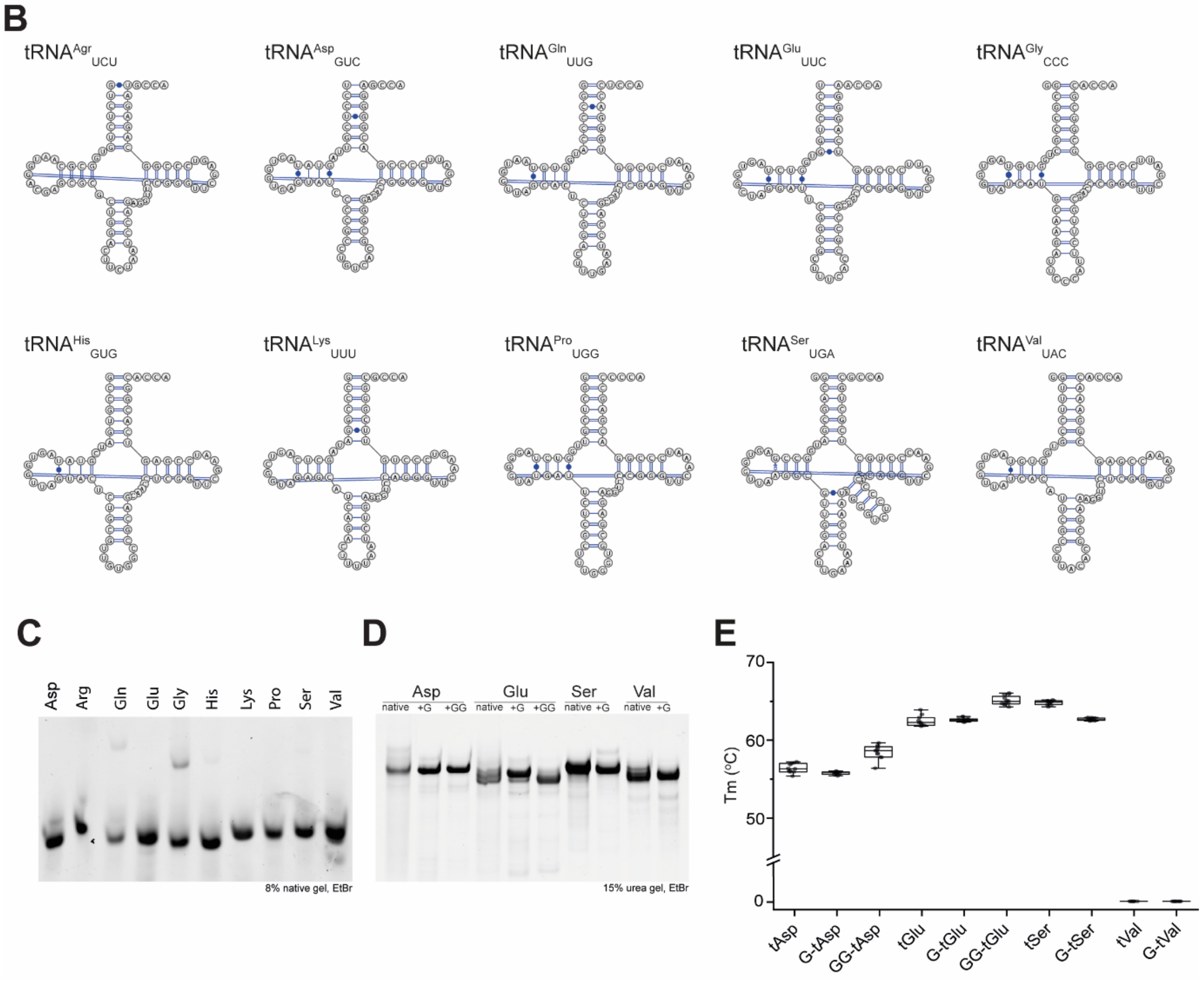

Appendix Figure S1. Sequences and predicted 2D structures of tRNAs used in this study. (**A**) Sequences of *in vitro* transcribed tRNAs. The additionally introduced Gs at 5'end of the sequence are shown in bold. The naturally occurred pseudouridine sites are highlighted in color (Ψ_{13} orange, $\Psi_{27/28}$ blue, Ψ_{39} green, Ψ_{54} yellow, Ψ_{55} red). (**B**) Predicted 2D structures of *in vitro* transcribed tRNAs. The interaction of G19-C56 is shown with a blue line while base pairings in each stem are indicated. (**C**) A native gel showing the mobility of each tRNA. (**D**) A urea gel showing the produced tRNA quality. (**E**) Melting temperatures (Tm) of the produced tRNAs.

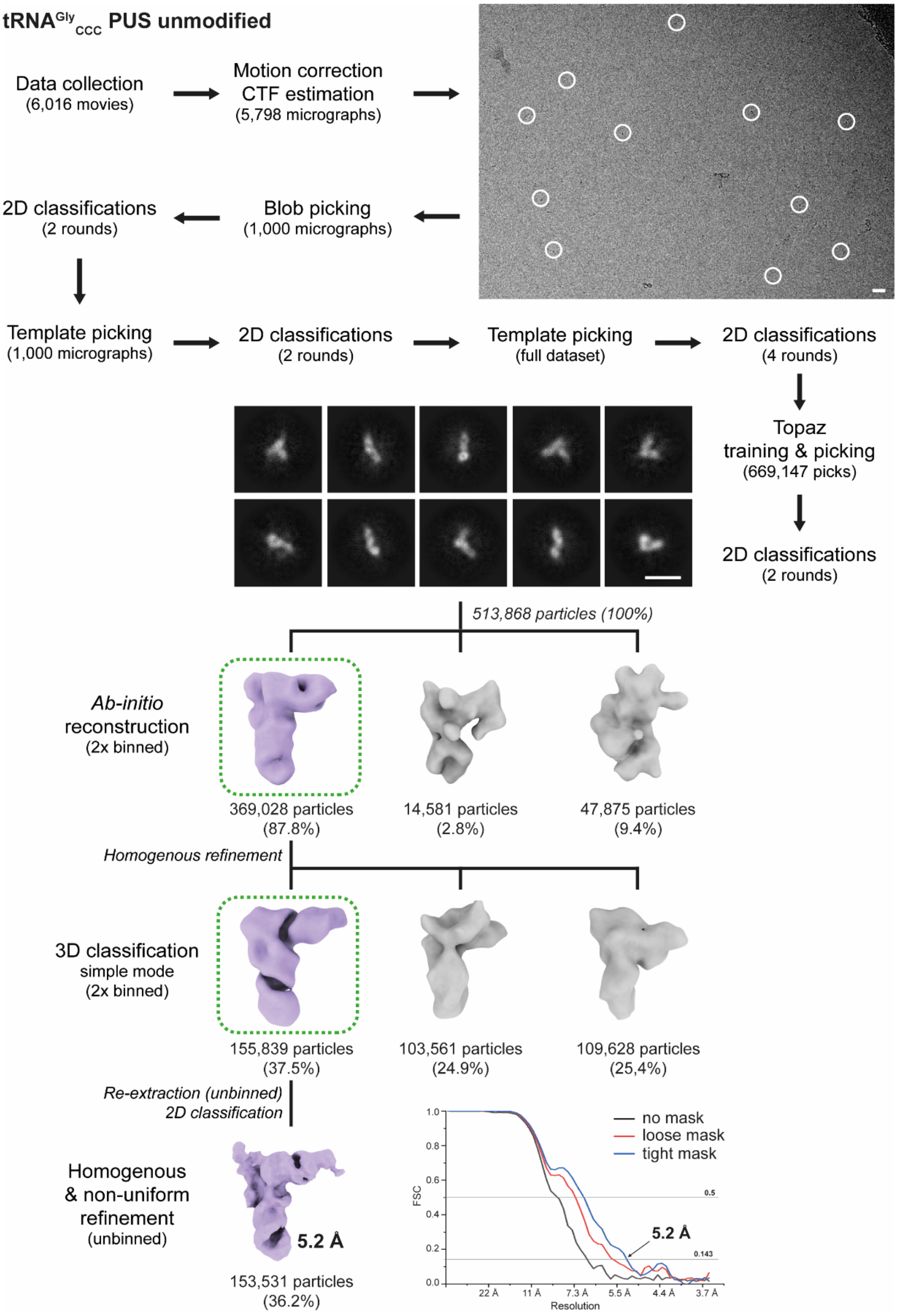

Appendix Figure S2. Cryo-EM reconstruction pipeline for unmodified tRNA^{Gly}$_{CCC}$. A representative micrograph is shown (top right) together with indicated positions of the finally selected particles (white circles); scalebar = 100 Å. Representative 2D classes, ab-initio classes and further steps of 3D refinement are shown in the bottom part of the figure. Absolute numbers and percentages of particles are listed and the Fourier Shell Correlation blot (FSC) blot of the final reconstruction, highlighting the nominal resolution at FSC=0.143.

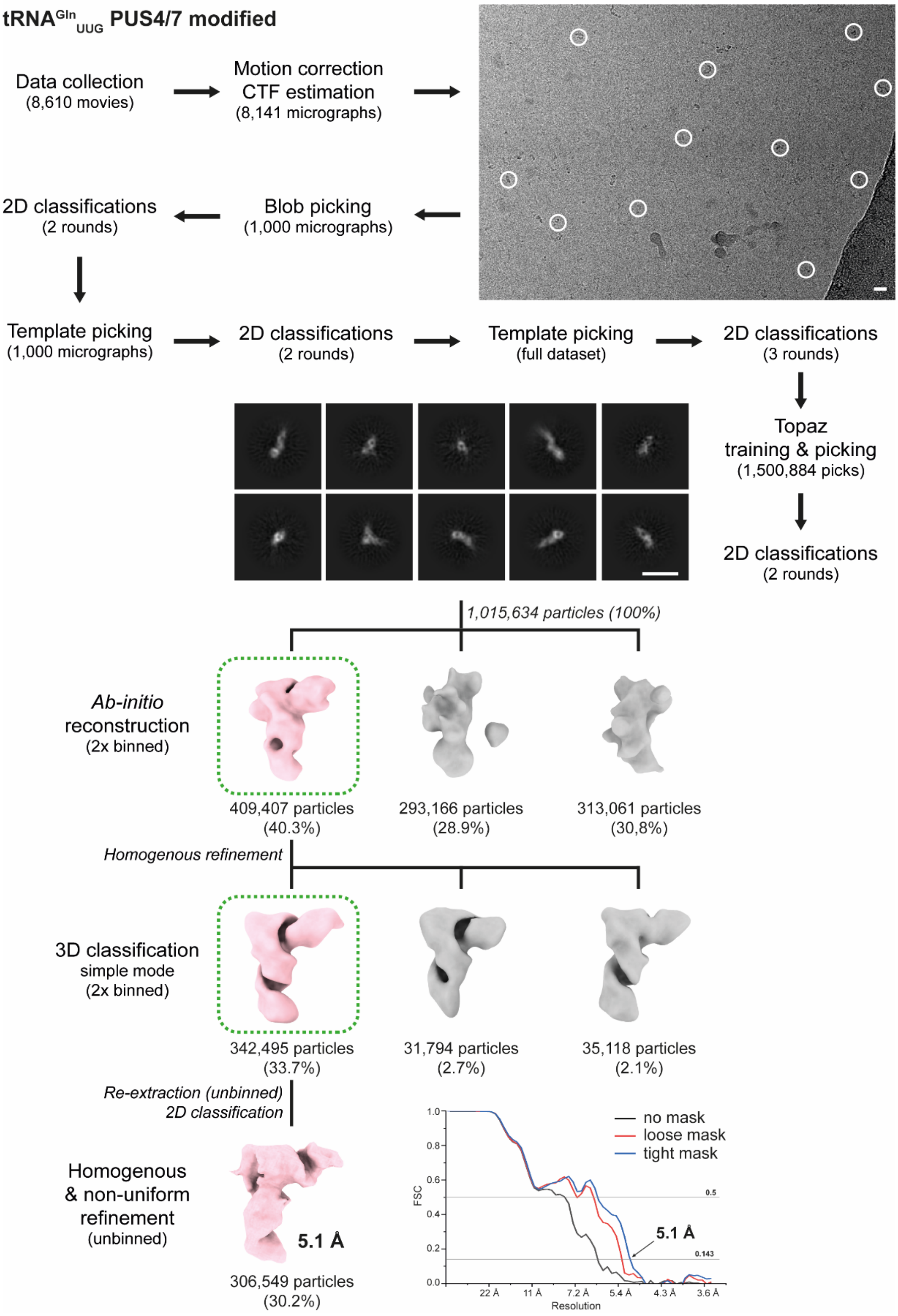

Appendix Figure S3. Cryo-EM reconstruction pipeline for PUS 4/7 modified tRNA$^{Gln}_{UUG}$. A representative micrograph is shown (top right) together with indicated positions of the finally selected particles (white circles); scalebar = 100 Å. Representative 2D classes, ab-initio classes and further steps of 3D refinement are shown in the bottom part of the figure. Absolute numbers and percentages of particles are listed and the Fourier Shell Correlation blot (FSC) blot of the final reconstruction, highlighting the nominal resolution at FSC=0.143.

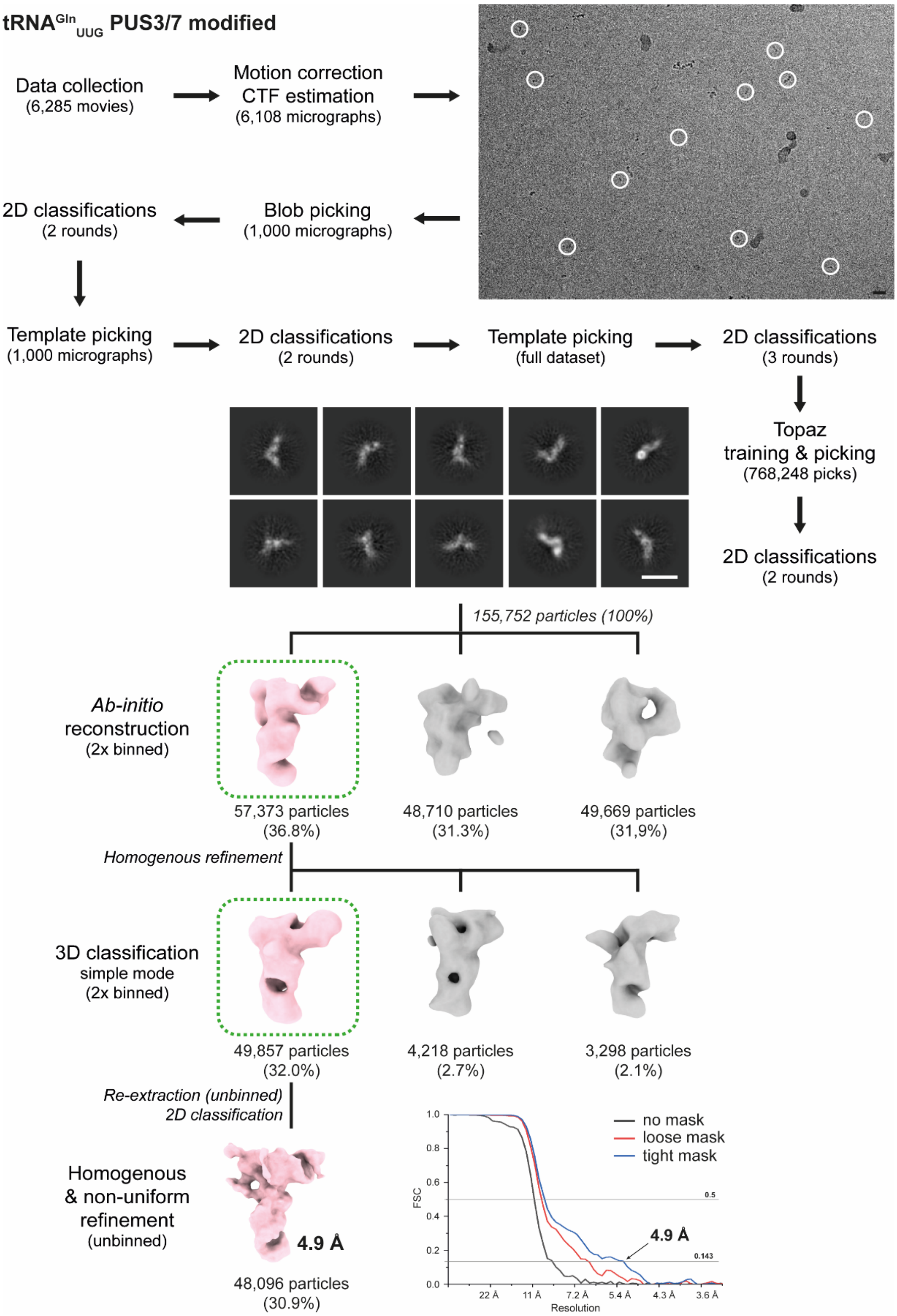

Appendix Figure S4. Cryo-EM reconstruction pipeline for PUS3/7 modified tRNA$^{Gln}_{UUG}$. A representative micrograph is shown (top right) together with indicated positions of the finally selected particles (white circles); scalebar = 100 Å. Representative 2D classes, ab-initio classes and further steps of 3D refinement are shown in the bottom part of the figure. Absolute numbers and percentages of particles are listed and the Fourier Shell Correlation blot (FSC) blot of the final reconstruction, highlighting the nominal resolution at FSC=0.143.

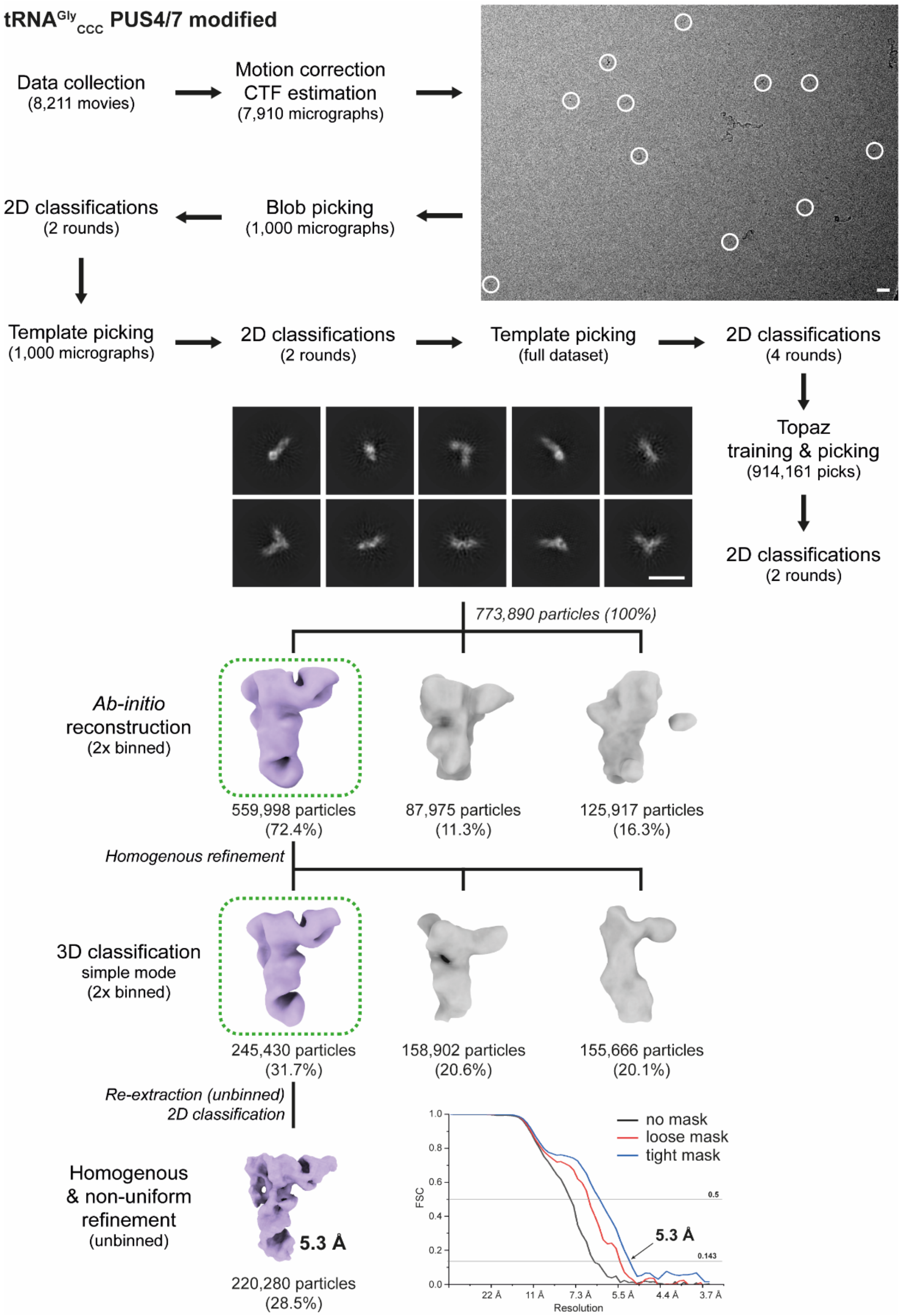

Appendix Figure S5. Cryo-EM reconstruction pipeline for PUS4/7 modified tRNA^{Gly}$_{CCC}$. A representative micrograph is shown (top right) together with indicated positions of the finally selected particles (white circles); scalebar = 100 Å. Representative 2D classes, ab-initio classes and further steps of 3D refinement are shown in the bottom part of the figure. Absolute numbers and percentages of particles are listed and the Fourier Shell Correlation blot (FSC) blot of the final reconstruction, highlighting the nominal resolution at FSC=0.143.

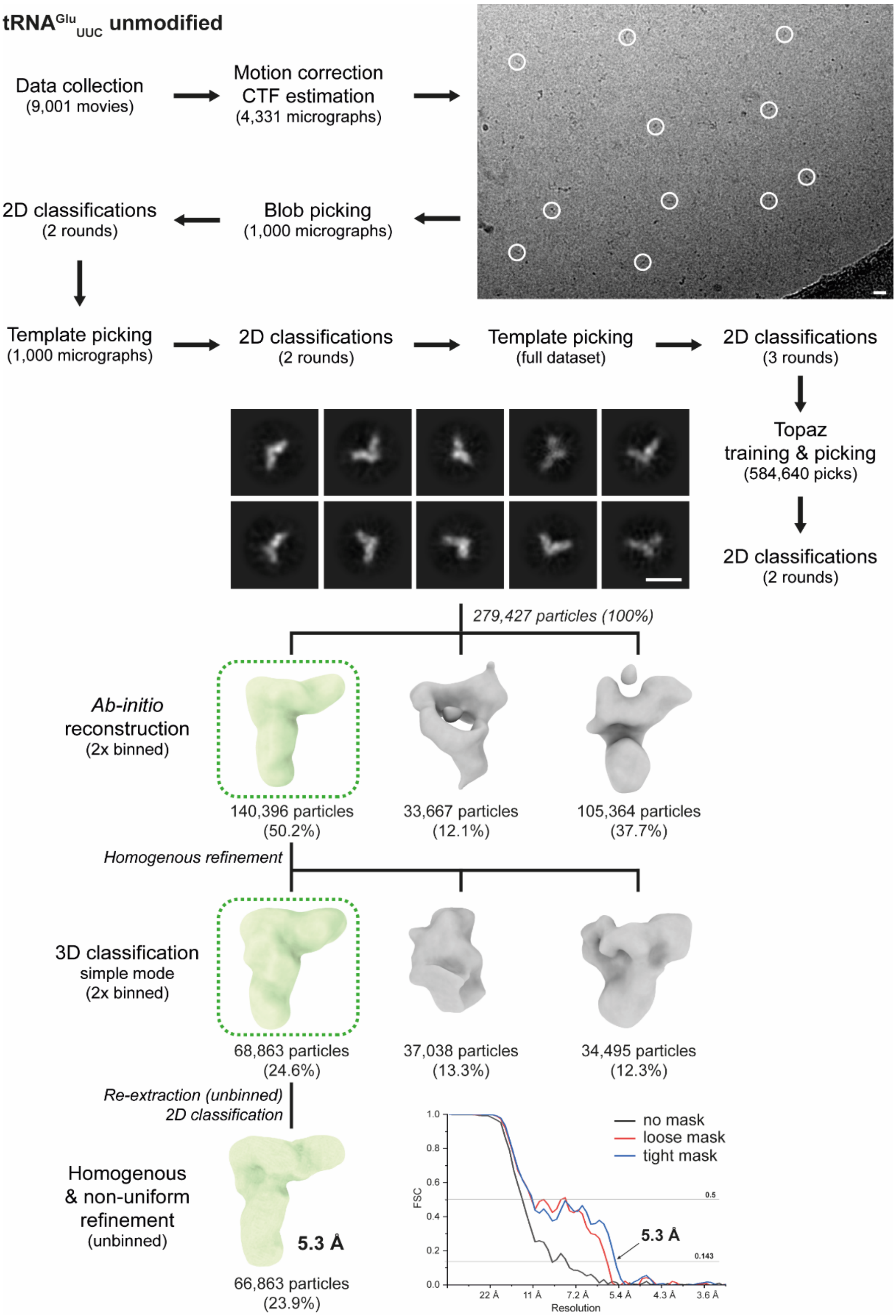

Appendix Figure S6. Cryo-EM reconstruction pipeline for unmodified tRNA^{Glu}$_{UUC}$. A representative micrograph is shown (top right) together with indicated positions of the finally selected particles (white circles); scalebar = 100 Å. Representative 2D classes, ab-initio classes and further steps of 3D refinement are shown in the bottom part of the figure. Absolute numbers and percentages of particles are listed and the Fourier Shell Correlation blot (FSC) blot of the final reconstruction, highlighting the nominal resolution at FSC=0.143.

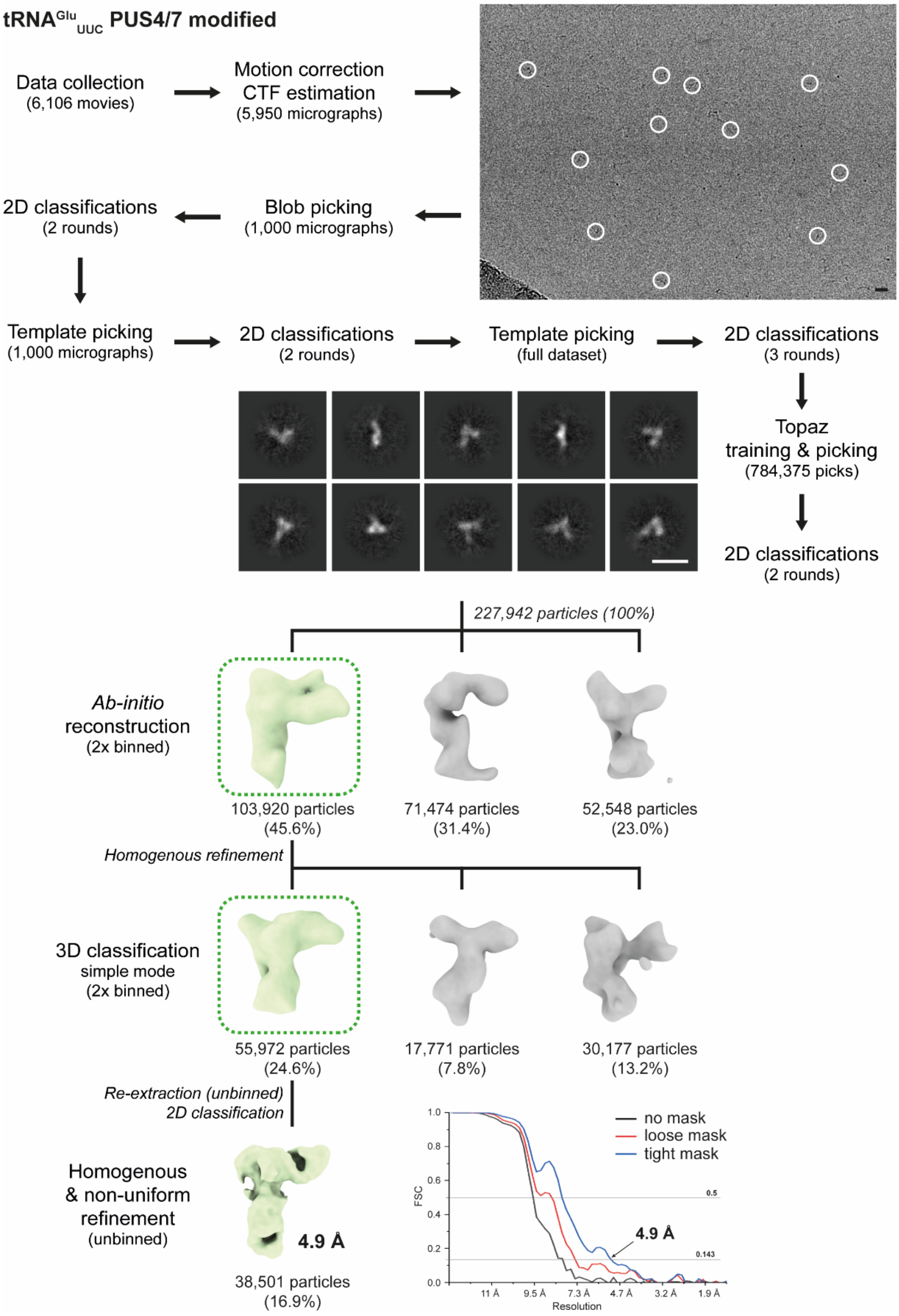

Appendix Figure S7. Cryo-EM reconstruction pipeline for PUS4/7 modified tRNA^{Glu}$_{UUC}$. A representative micrograph is shown (top right) together with indicated positions of the finally selected particles (white circles); scalebar = 100 Å. Representative 2D classes, ab-initio classes and further steps of 3D refinement are shown in the bottom part of the figure. Absolute numbers and percentages of particles are listed and the Fourier Shell Correlation blot (FSC) blot of the final reconstruction, highlighting the nominal resolution at FSC=0.143.

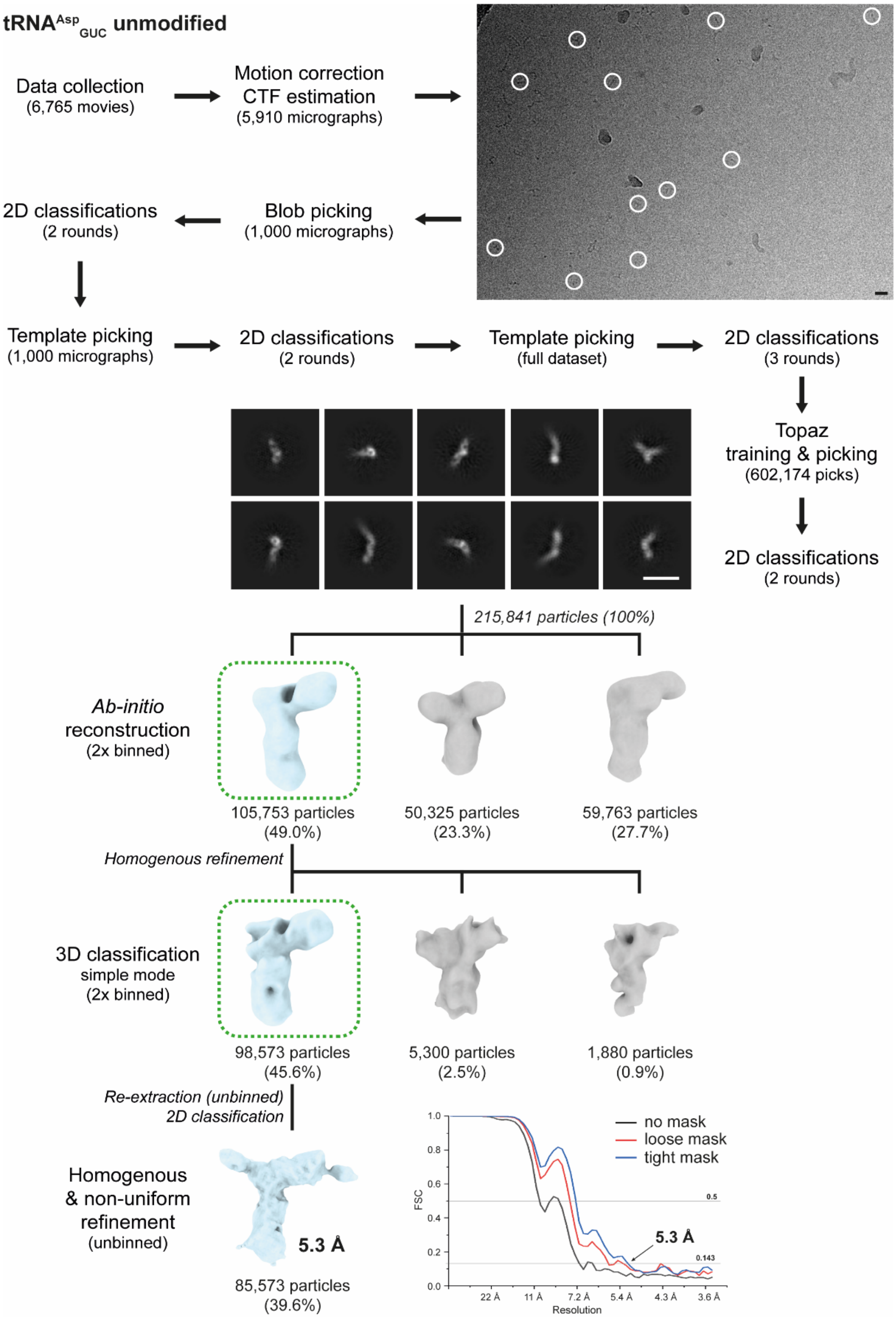

Appendix Figure S8. Cryo-EM reconstruction pipeline for unmodified tRNA$^{Asp}_{GUC}$. A representative micrograph is shown (top right) together with indicated positions of the finally selected particles (white circles); scalebar = 100 Å. Representative 2D classes, ab-initio classes and further steps of 3D refinement are shown in the bottom part of the figure. Absolute numbers and percentages of particles are listed and the Fourier Shell Correlation blot (FSC) blot of the final reconstruction, highlighting the nominal resolution at FSC=0.143.

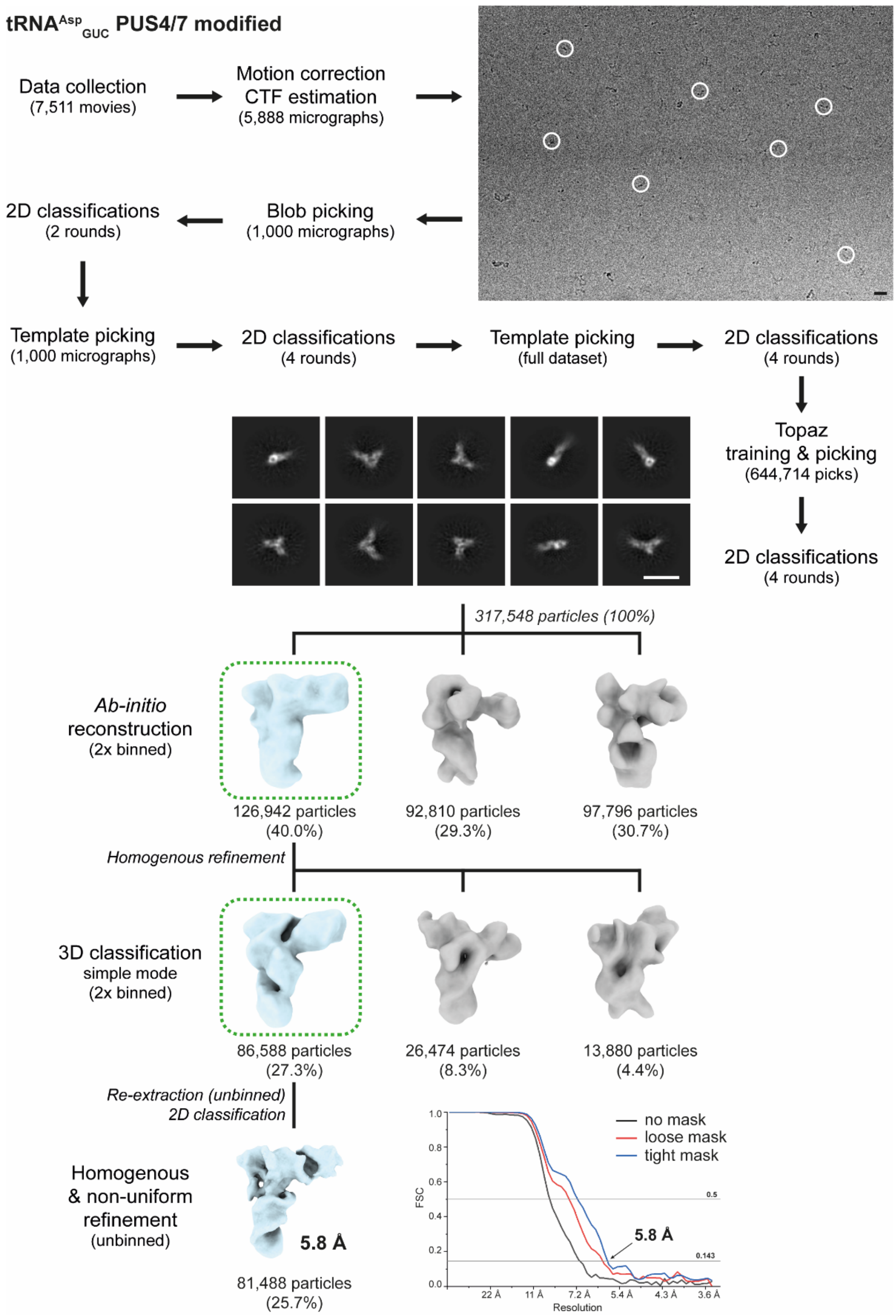

Appendix Figure S9. Cryo-EM reconstruction pipeline for PUS4/7 modified tRNA$^{Asp}_{GUC}$. A representative micrograph is shown (top right) together with indicated positions of the finally selected particles (white circles); scalebar = 100 Å. Representative 2D classes, ab-initio classes and further steps of 3D refinement are shown in the bottom part of the figure. Absolute numbers and percentages of particles are listed and the Fourier Shell Correlation blot (FSC) blot of the final reconstruction, highlighting the nominal resolution at FSC=0.143.

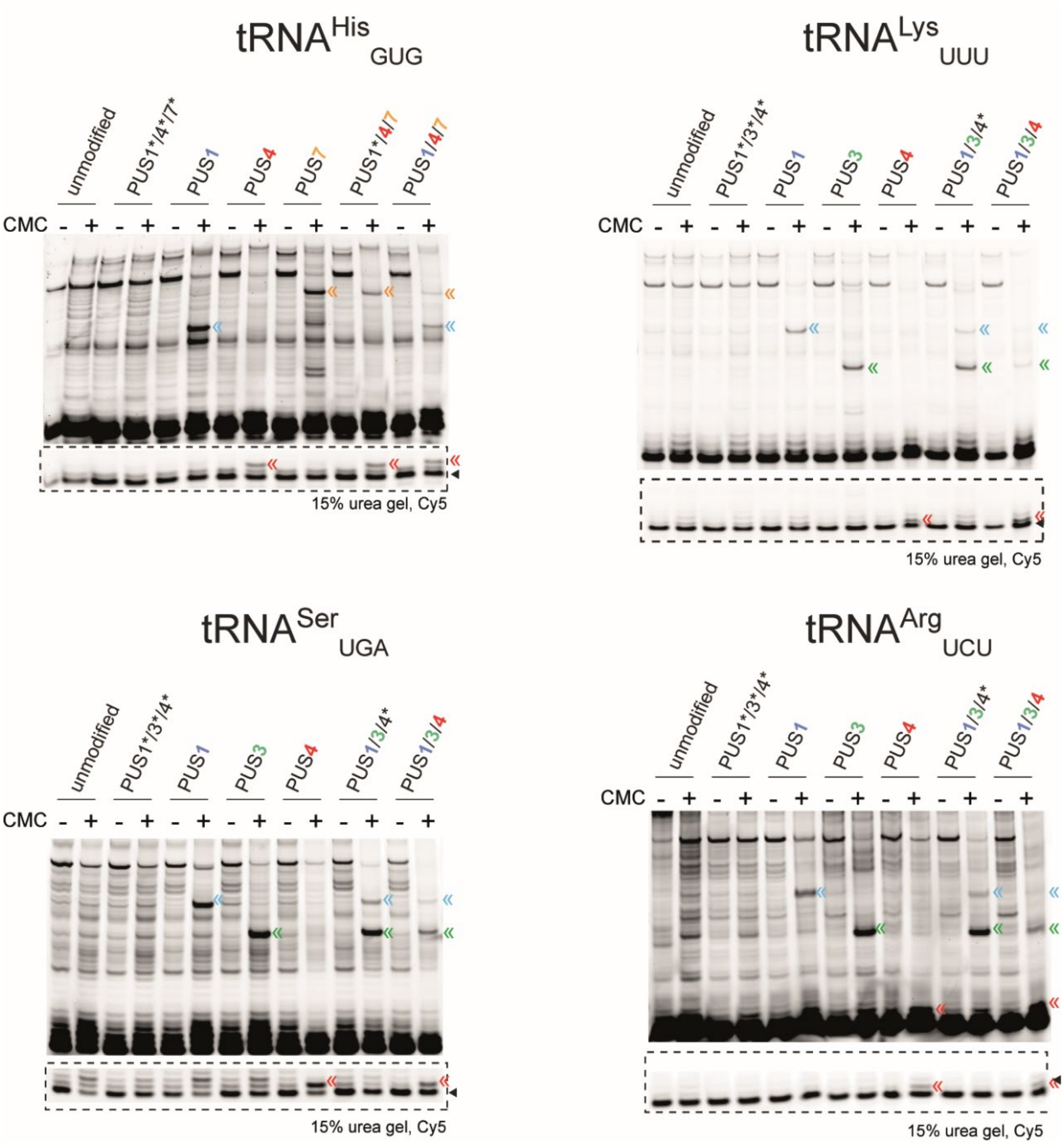

Appendix Figure S10. Detection of PUS-dependent Ψ formation on $tRNA^{His}_{GUG}$, $tRNA^{Lys}_{UUU}$, $tRNA^{Ser}_{UGA}$, $tRNA^{Arg}_{UCU}$. The reverse-transcribed cDNA products were resolved in a 15% urea gel and the CMC-Ψ mediated short cDNAs are indicated by double-arrows (Ψ_{13} orange, $\Psi_{27/28}$ blue, Ψ_{39} green, Ψ_{55} red). The signal for each primer (indicated by triangles) is obtained from a short exposure shown in the dash lined box.

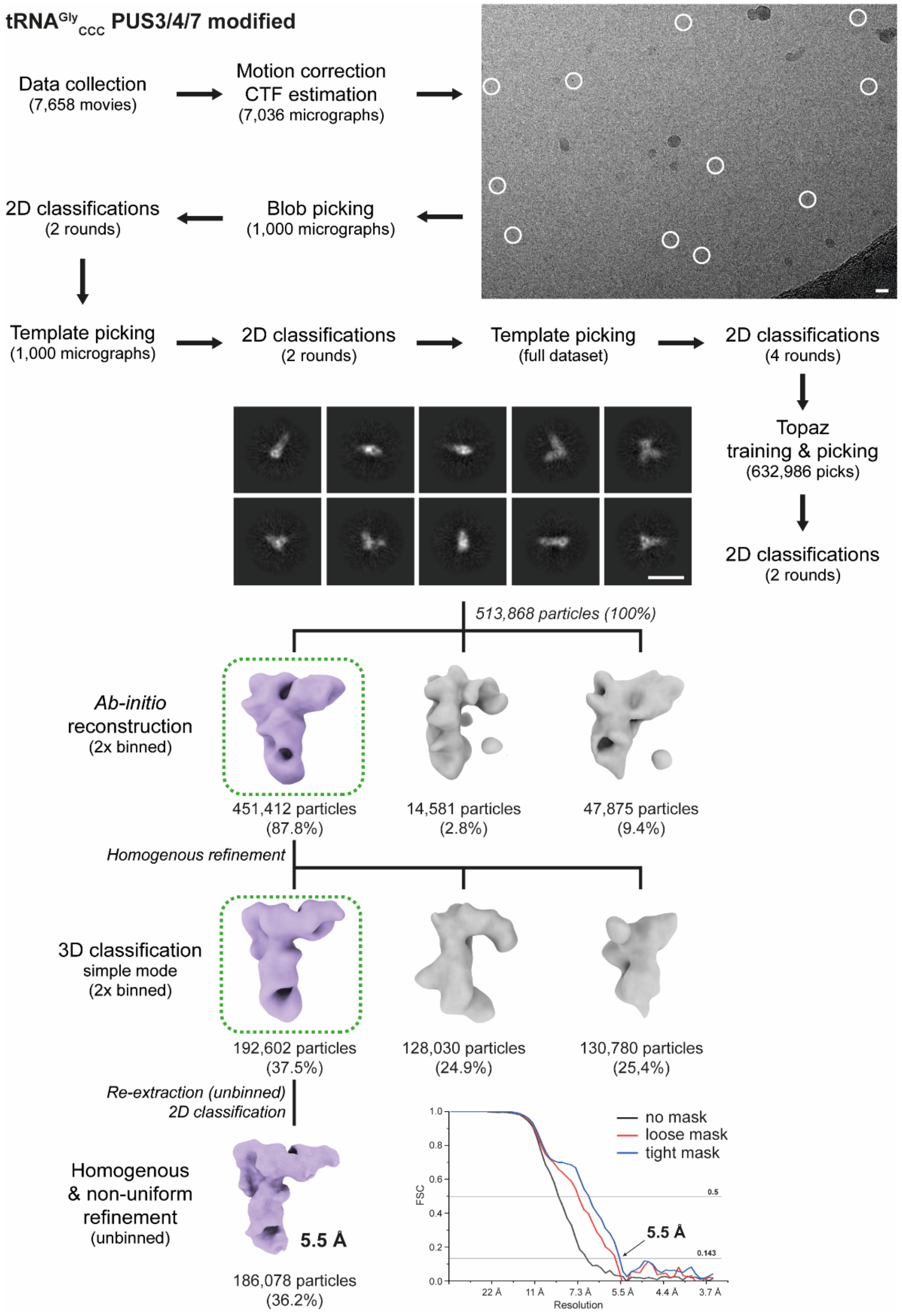

Appendix Figure S11. Cryo-EM reconstruction pipeline for PUS3/4/7 modified tRNA^{Gly}$_{CCC}$. A representative micrograph is shown (top right) together with indicated positions of the finally selected particles (white circles); scalebar = 100 Å. Representative 2D classes, ab-initio classes and further steps of 3D refinement are shown in the bottom part of the figure. Absolute numbers and percentages of particles are listed and the Fourier Shell Correlation blot (FSC) blot of the final reconstruction, highlighting the nominal resolution at FSC=0.143.

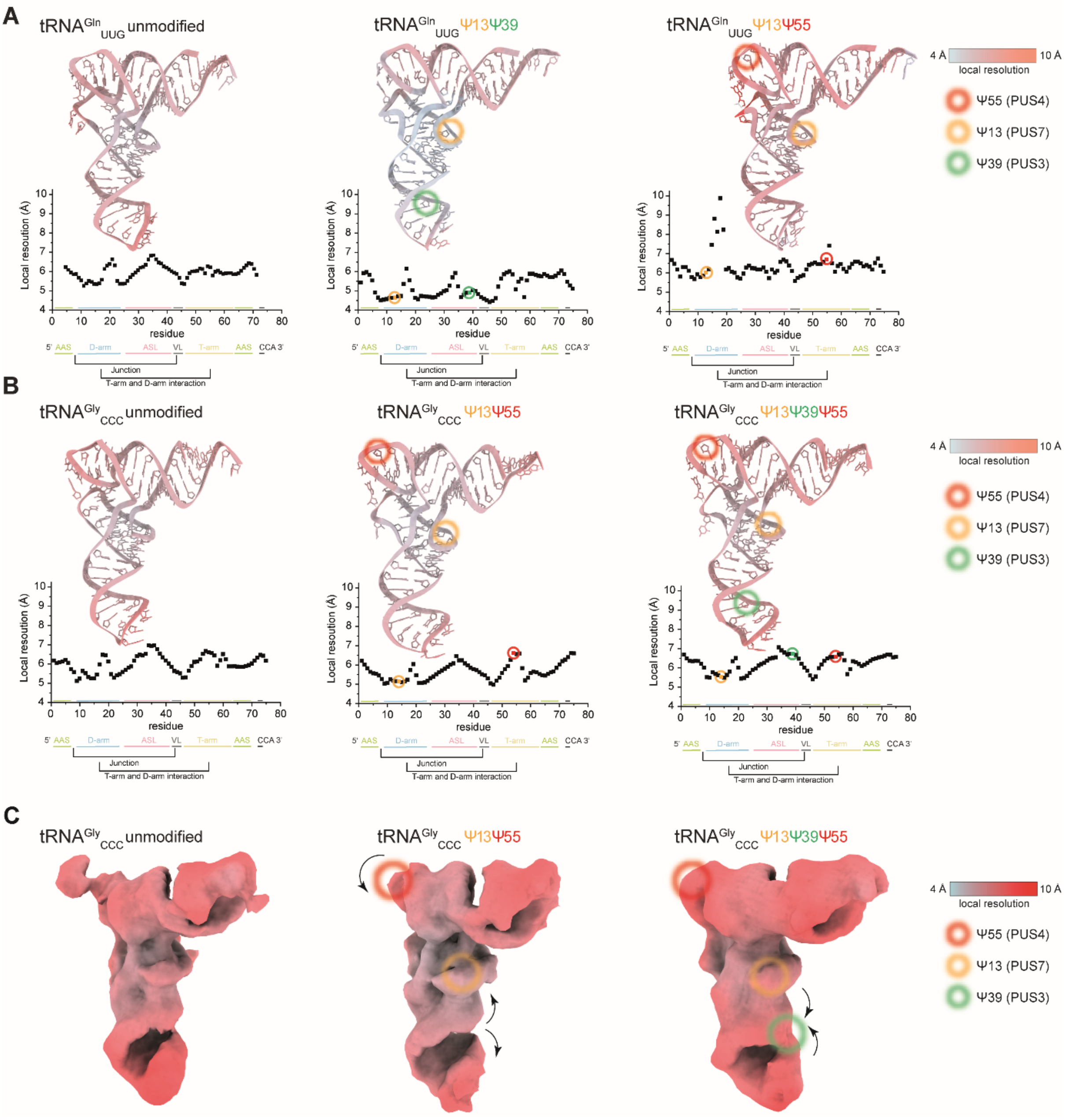

Appendix Figure S12. Local resolution in regions around the respective modification sites of tRNAs. (**A-B**) Atomic models colored by local resolution estimations for tRNA^{Gln}$_{UUG}$ (**A**) and tRNA^{Gly}$_{CCC}$ (**B**). The relative resolution scale is shown in the inset. Below each model, a plot shows local resolution estimation for each residue. Introduced Ψ sites are highlighted by circles ($Ψ_{13}$ orange, $Ψ_{39}$ green, $Ψ_{55}$ red). tRNA domains are as indicated: acceptor arm (AAS, green); T-arm (yellow); D-arm (blue); variable loop (VL, grey); anticodon arm (ASL, pink). (**C**) Local resolution estimations for tRNA^{Gly}$_{CCC}$. The relative resolution scale is shown in the inset. Ψ sites are highlighted by circles ($Ψ_{13}$ orange, $Ψ_{39}$ green, $Ψ_{55}$ red). The arrows indicate the local conformational changes. All cryo-EM maps are contoured to RMSD = 8.
