# Supplementary material for: Determining the effects of pseudouridine incorporation on human tRNAs
Source: EMBO J. 2025 Apr 29;44(13):3553–85. doi: 10.1038/s44318-025-00443-y (PMC12217144; doi:10.1038/s44318-025-00443-y)
Supplement: Supplementary file 3 — Movie EV1 [file 44318_2025_443_MOESM3_ESM.zip › Legend_MovieEV1.docx]

**Movie EV1. Movie of a representative simulation, highlighting the interaction between D- and T-arm.**
