# Supplementary material for: Determining the effects of pseudouridine incorporation on human tRNAs
Source: EMBO J. 2025 Apr 29;44(13):3553–85. doi: 10.1038/s44318-025-00443-y (PMC12217144; doi:10.1038/s44318-025-00443-y)
Supplement: Supplementary file 4 — Movie EV2 [file 44318_2025_443_MOESM4_ESM.zip › Legend_MovieEV2.docx]

**Movie EV2. Movie of a representative simulation, highlighting the presence and absence of canonical and non-canonical base pairs.**
