# Supplementary material for: Determining the effects of pseudouridine incorporation on human tRNAs
Source: EMBO J. 2025 Apr 29;44(13):3553–85. doi: 10.1038/s44318-025-00443-y (PMC12217144; doi:10.1038/s44318-025-00443-y)
Supplement: Supplementary file 5 — Movie EV3 [file 44318_2025_443_MOESM5_ESM.zip › Legend_MovieEV3.docx]

**Movie EV3. Movie showing the structural changes induced by the introduction of Ψ at different position in human tRNAGlnUUG.**
