# Supplementary figures and images for: Determining the effects of pseudouridine incorporation on human tRNAs
Source: EMBO J. 2025 Apr 29;44(13):3553–85. doi: 10.1038/s44318-025-00443-y (PMC12217144; doi:10.1038/s44318-025-00443-y)

EMBOJ-2025-120166-T_SourceDataForFigure1A


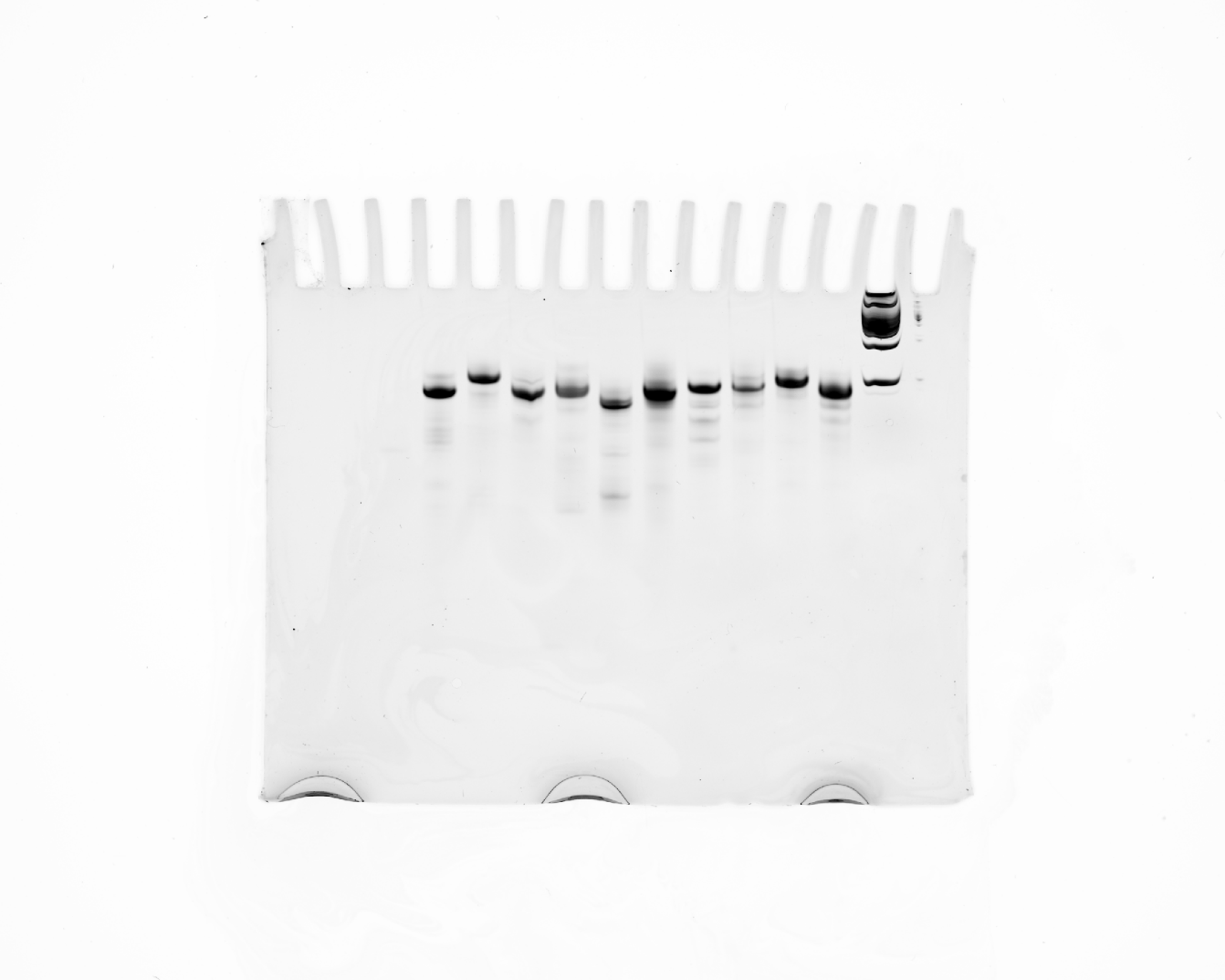

Supplement: Supplementary file 7 — Source data Fig. 1 [file 44318_2025_443_MOESM7_ESM.zip › EMBOJ-2025-120166-T-SourceData_uncroppedgels_Figure1A.docx]

EMBOJ-2025-120166-T_SourceDataForFigure5A_left_and_middle


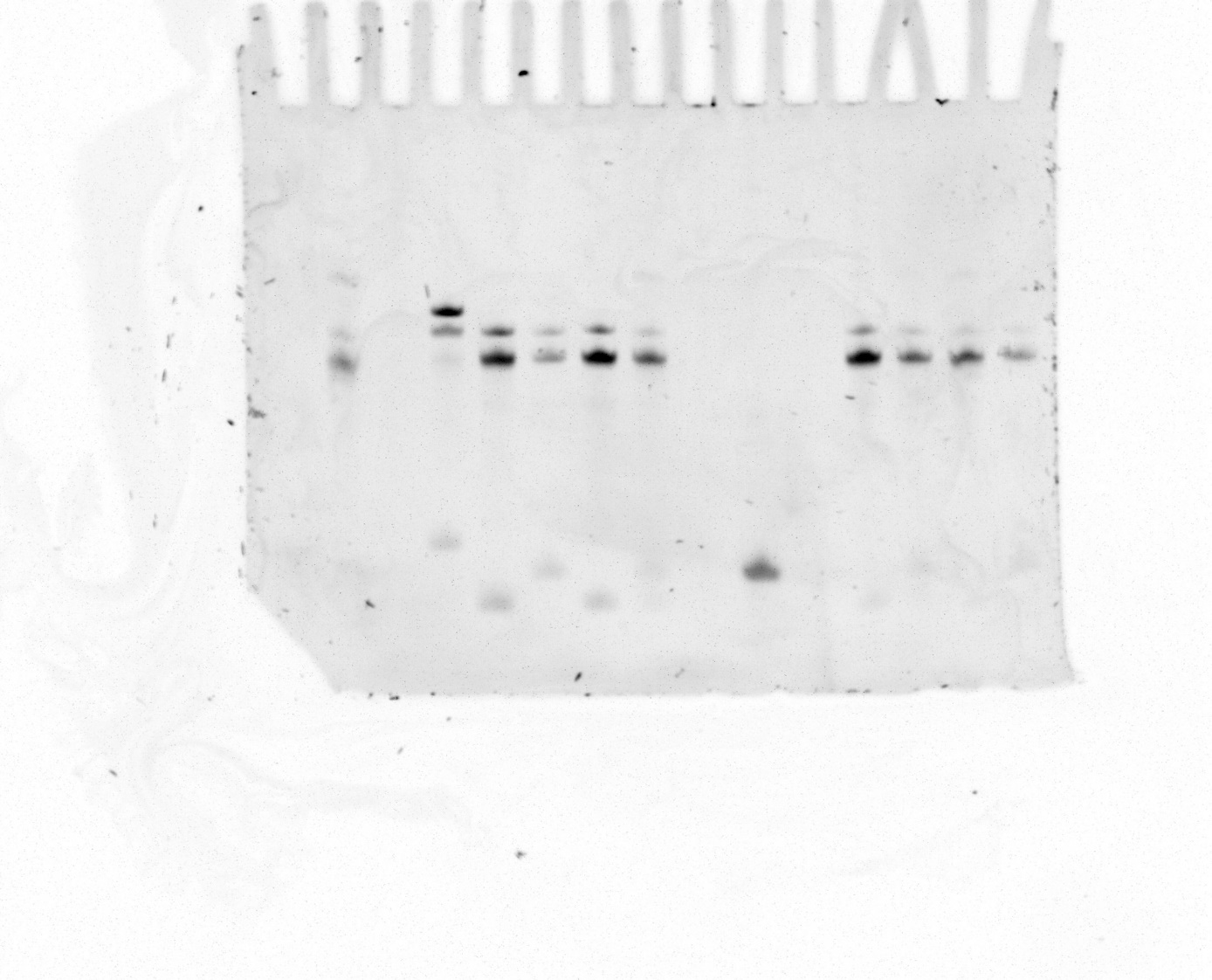


EMBOJ-2025-120166-T_SourceDataForFigure5A_right


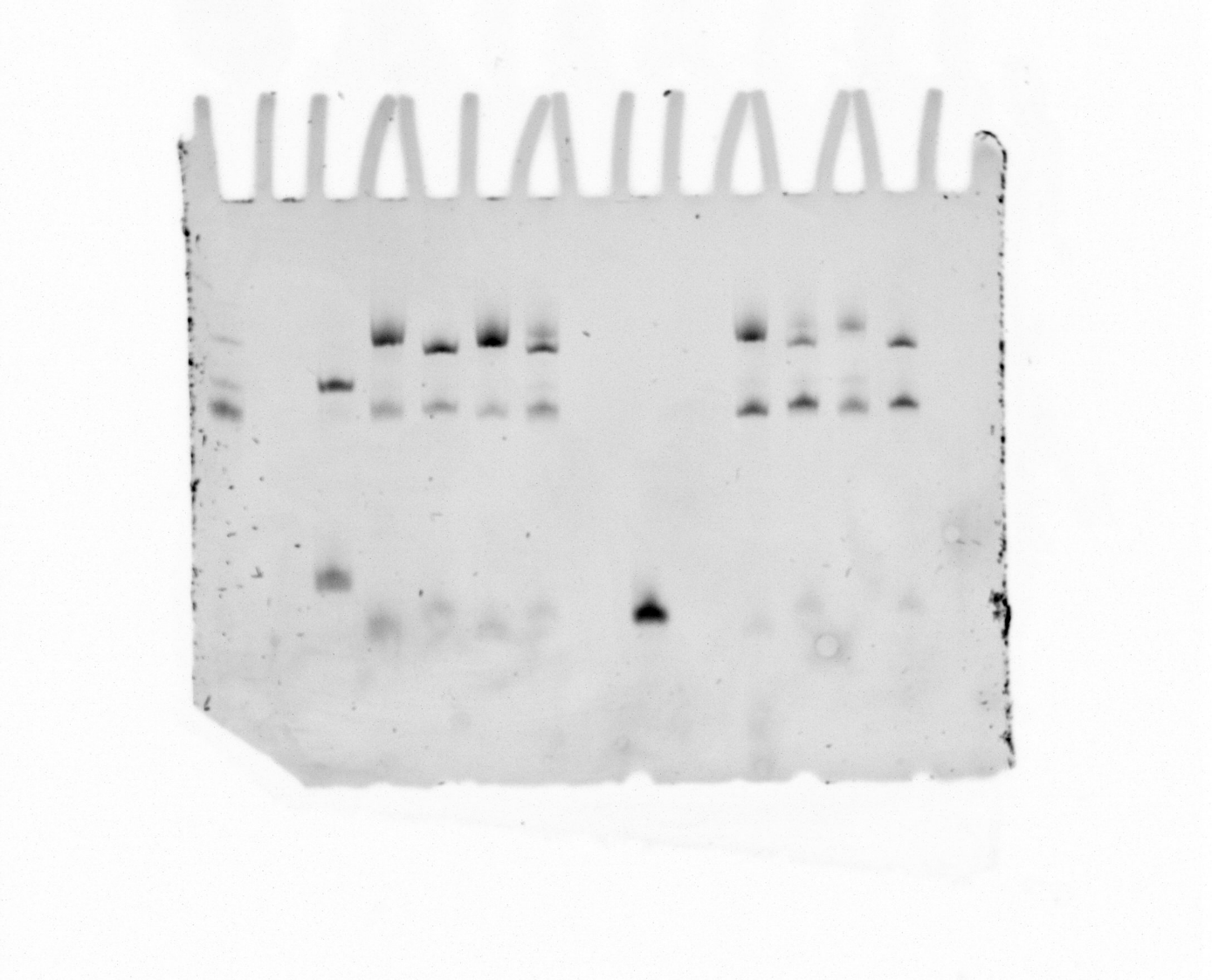

Supplement: Supplementary file 10 — Source data Fig. 5 [file 44318_2025_443_MOESM10_ESM.zip › EMBOJ-2025-120166-T-SourceData_uncroppedgels_Figure5.docx]

EMBOJ-2025-120166-T_SourceDataForAppendixFigureS1C


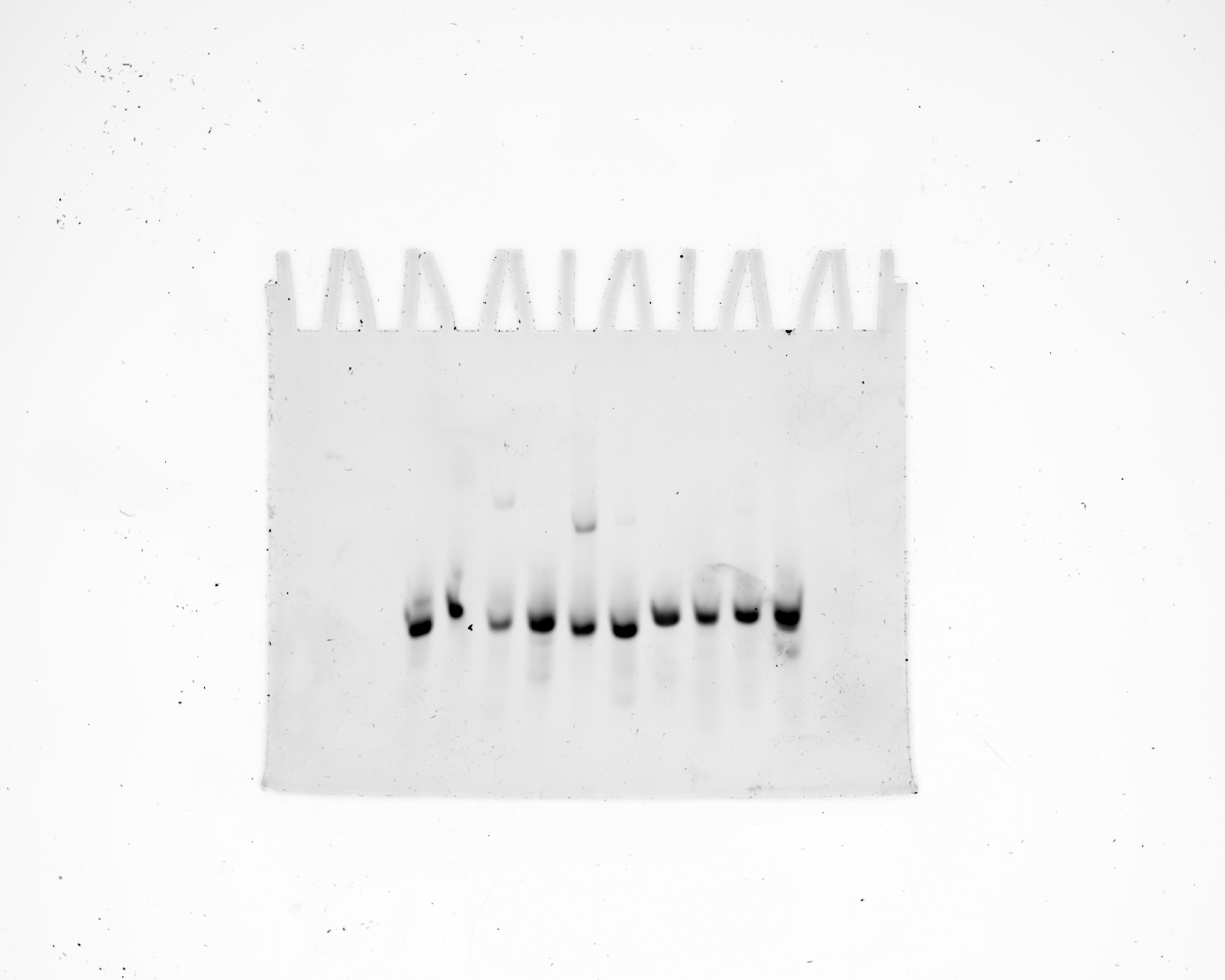


EMBOJ-2025-120166-T_SourceDataForAppendixFigureS1D


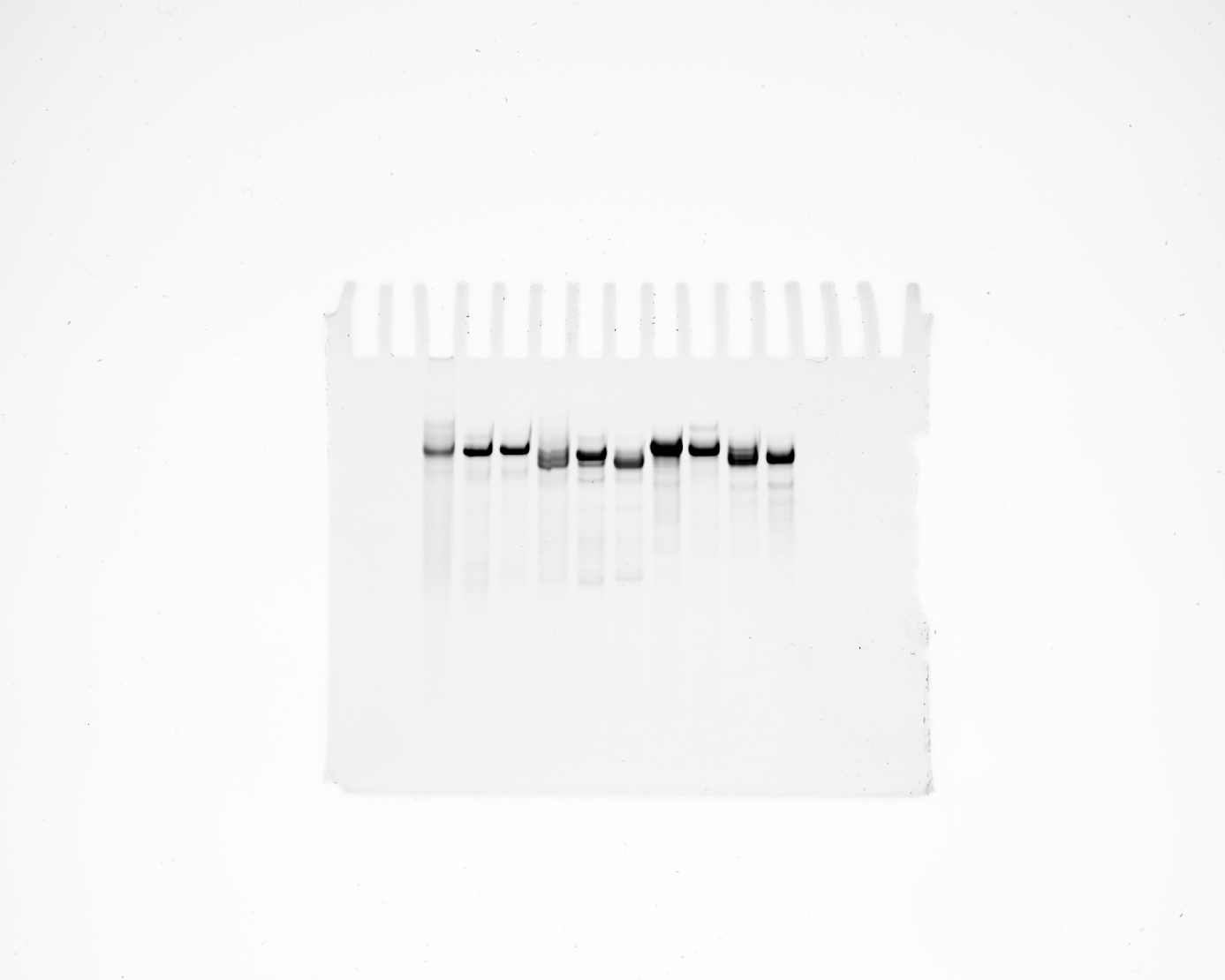

Supplement: Supplementary file 11 — EV and Appendix Figure Source Data [file 44318_2025_443_MOESM11_ESM.zip › EMBOJ-2025-120166-T-SourceData_uncroppedgels_AppendixFigureS1.docx]
