# Supplementary material for: Determining the effects of pseudouridine incorporation on human tRNAs
Source: EMBO J. 2025 Apr 29;44(13):3553–85. doi: 10.1038/s44318-025-00443-y (PMC12217144; doi:10.1038/s44318-025-00443-y)

EMBOJ-2025-120166-T_SourceDataForFigure2C_top


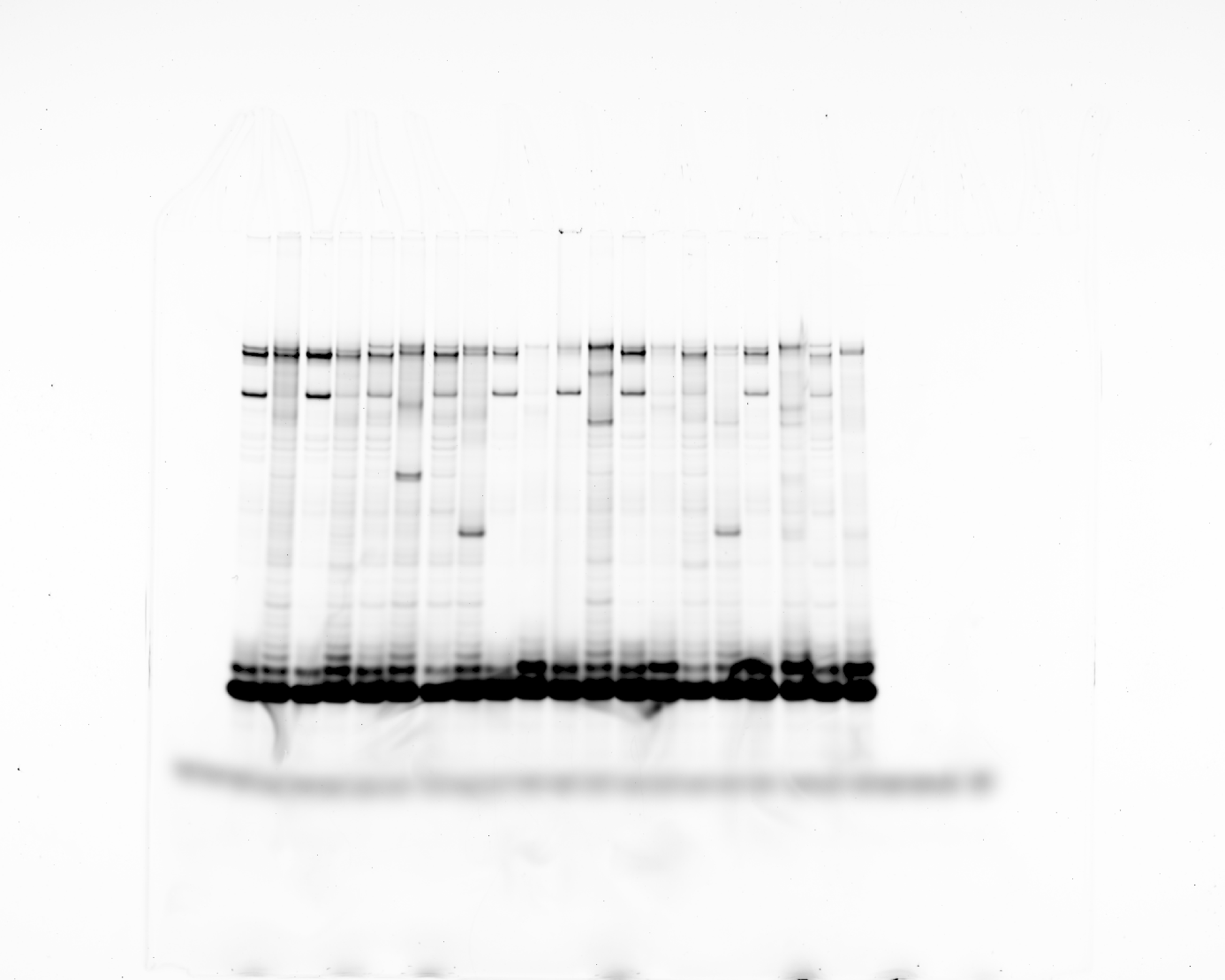


EMBOJ-2025-120166-T_SourceDataForFigure2C_bottom


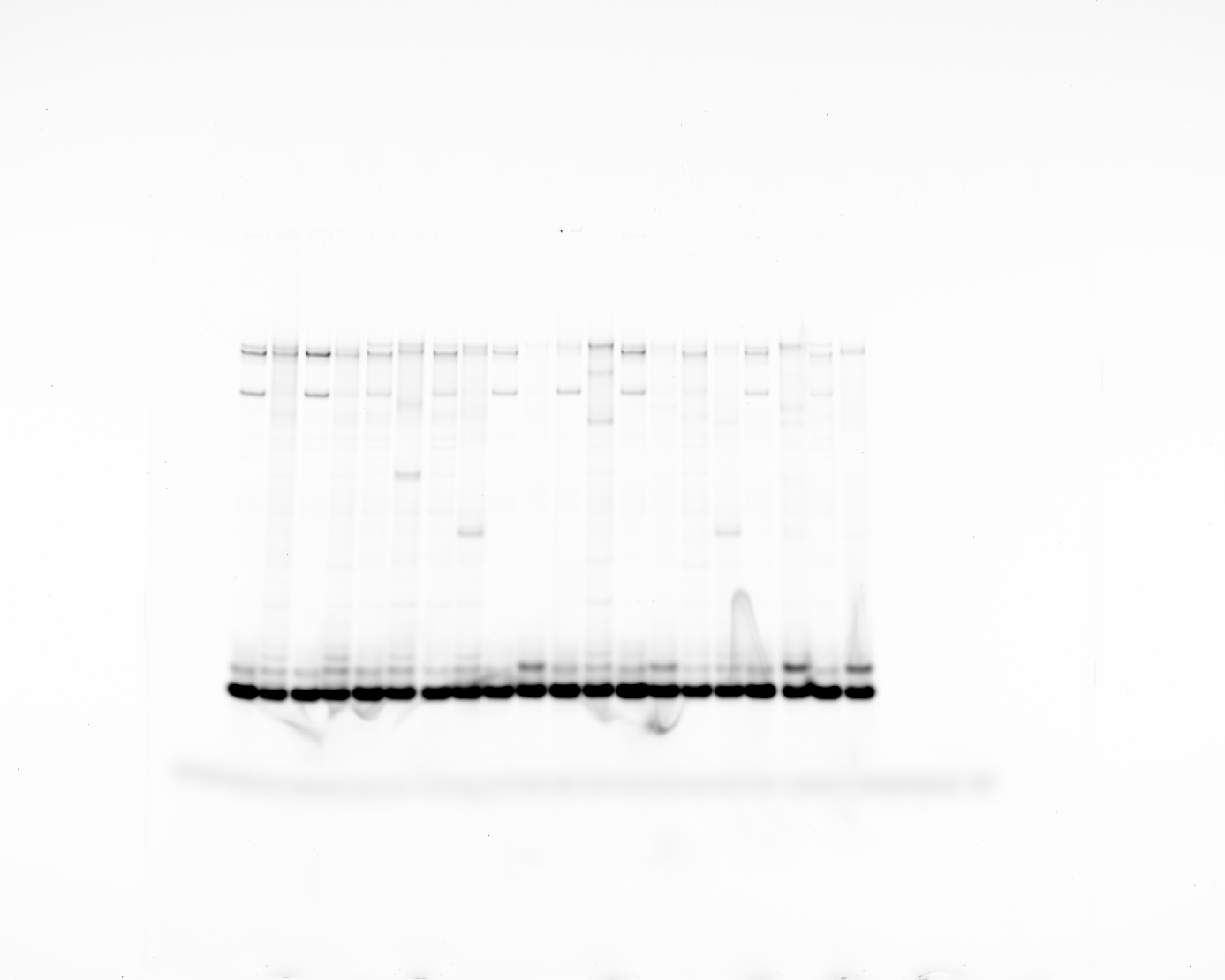


EMBOJ-2025-120166-T_SourceDataForFigure2G_left


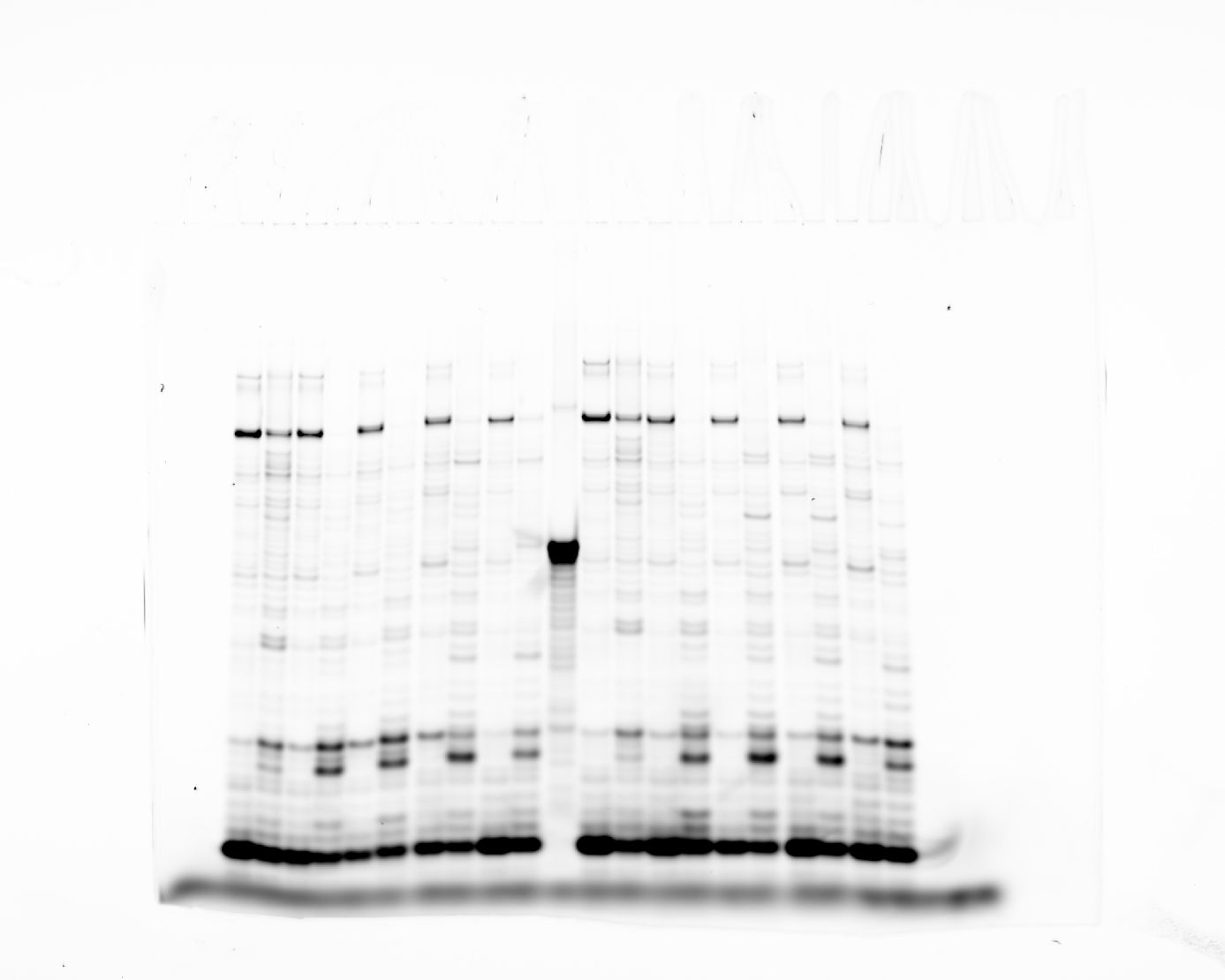


EMBOJ-2025-120166-T_SourceDataForFigure2G_middle


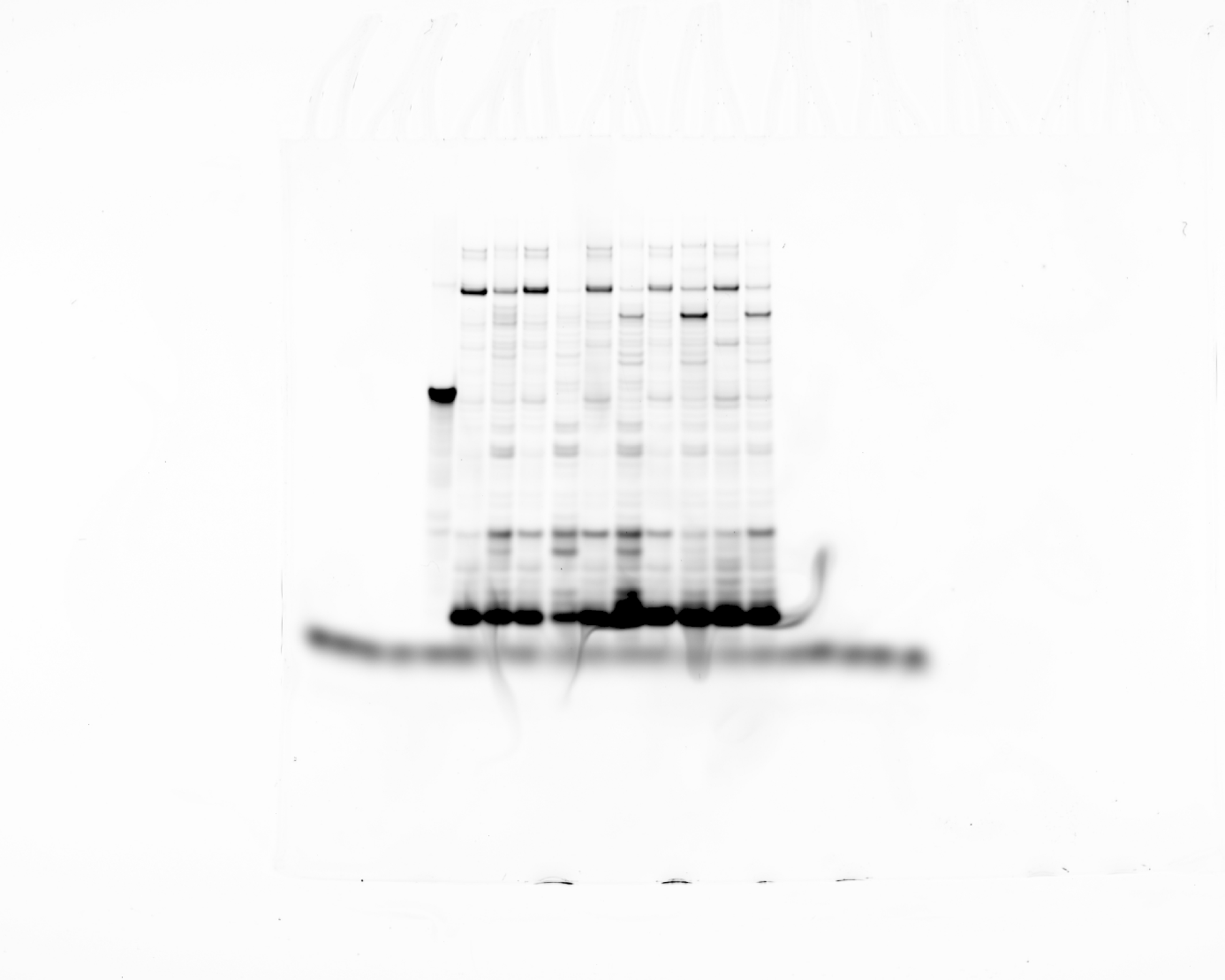


EMBOJ-2025-120166-T_SourceDataForFigure2G_right


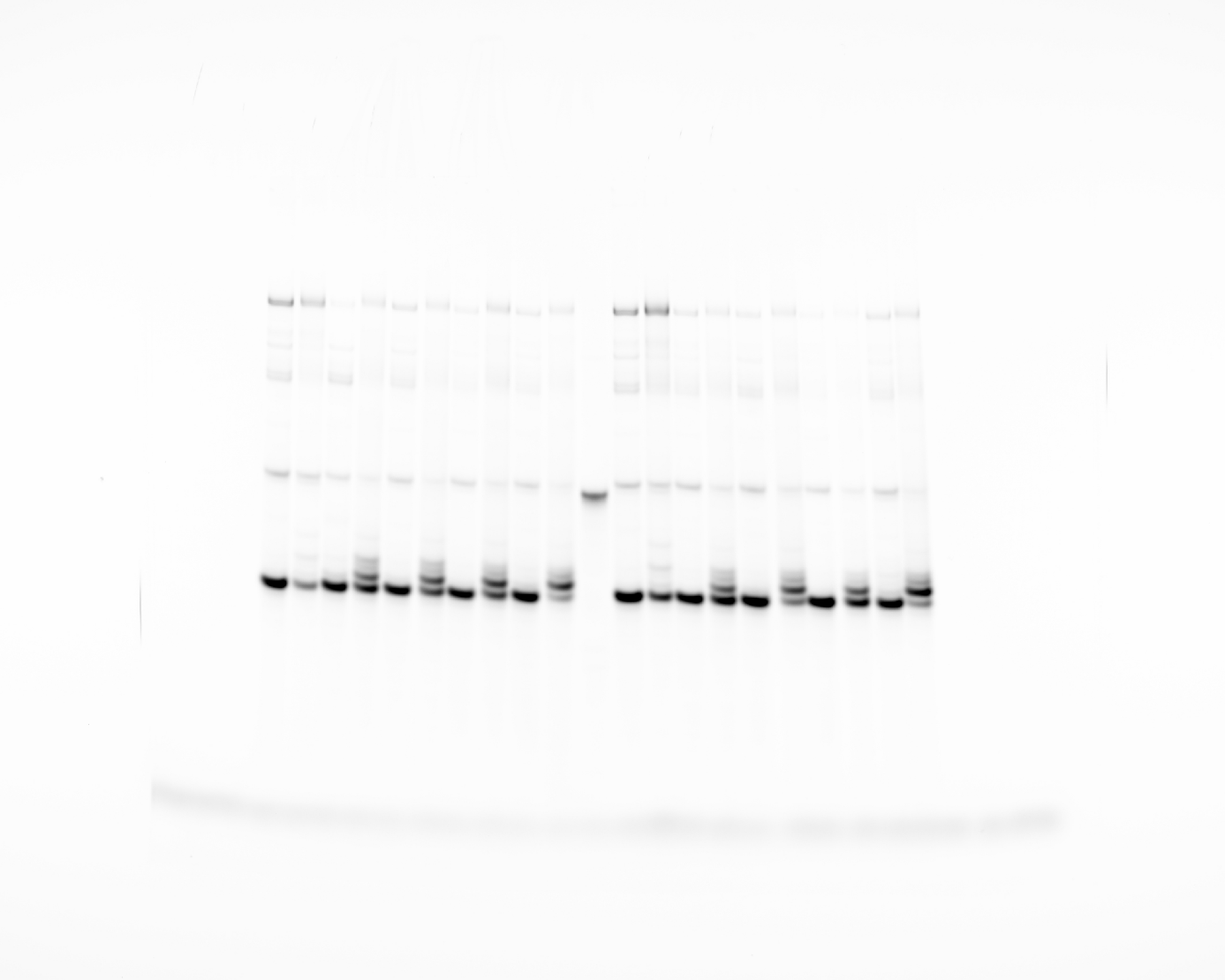

Supplement: Supplementary file 8 — Source data Fig. 2 [file 44318_2025_443_MOESM8_ESM.zip › EMBOJ-2025-120166-T-SourceData_uncroppedgels_Figure2C_2G.docx]
