# Supplementary material for: Determining the effects of pseudouridine incorporation on human tRNAs
Source: EMBO J. 2025 Apr 29;44(13):3553–85. doi: 10.1038/s44318-025-00443-y (PMC12217144; doi:10.1038/s44318-025-00443-y)

EMBOJ-2025-120166-T_SourceDataForAppendixFigureS11_His_top


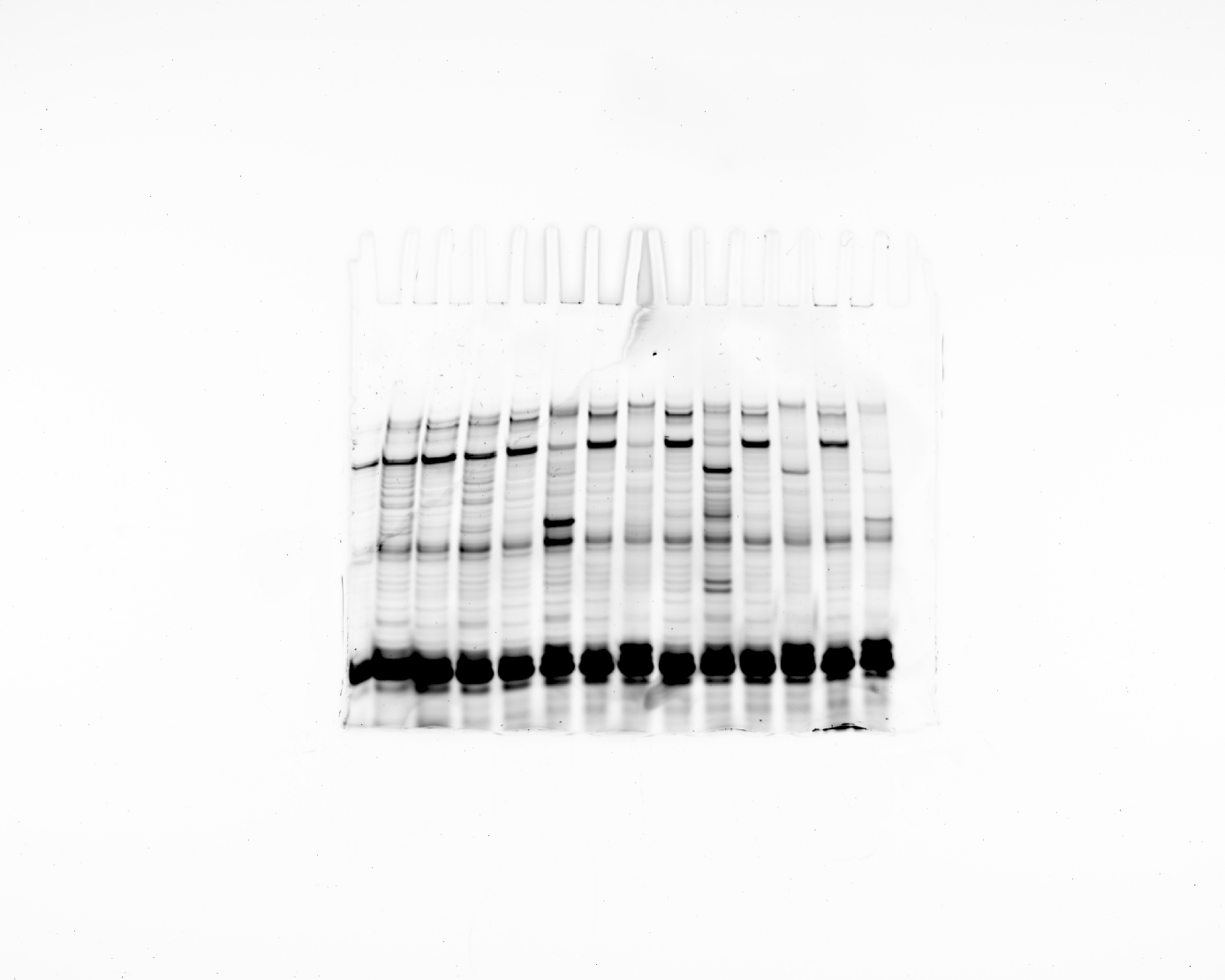


EMBOJ-2025-120166-T_SourceDataForAppendixFigureS11_His_bottom


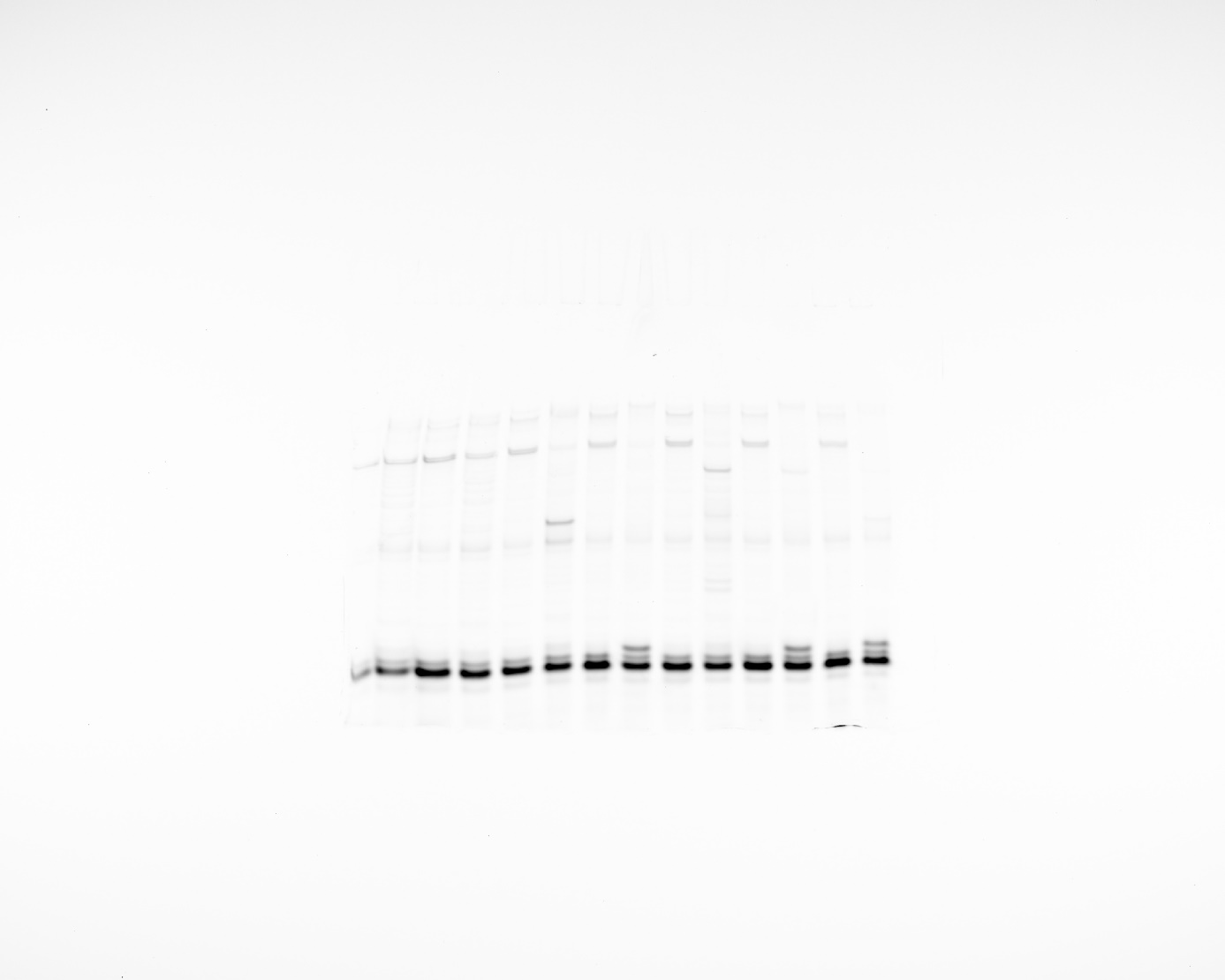


EMBOJ-2025-120166-T_SourceDataForAppendixFigureS11_Lys_top


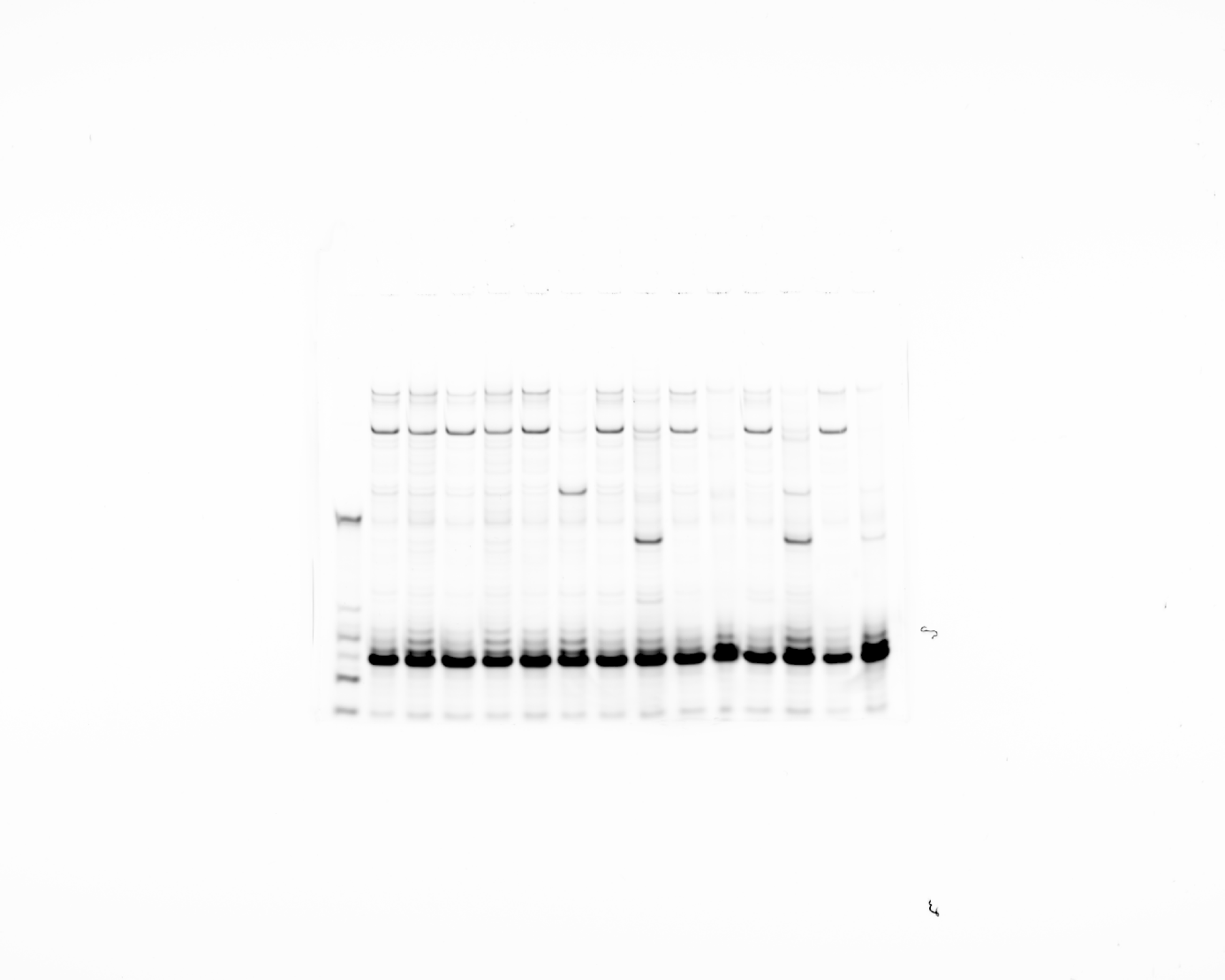


EMBOJ-2025-120166-T_SourceDataForAppendixFigureS11_Lys_bottom


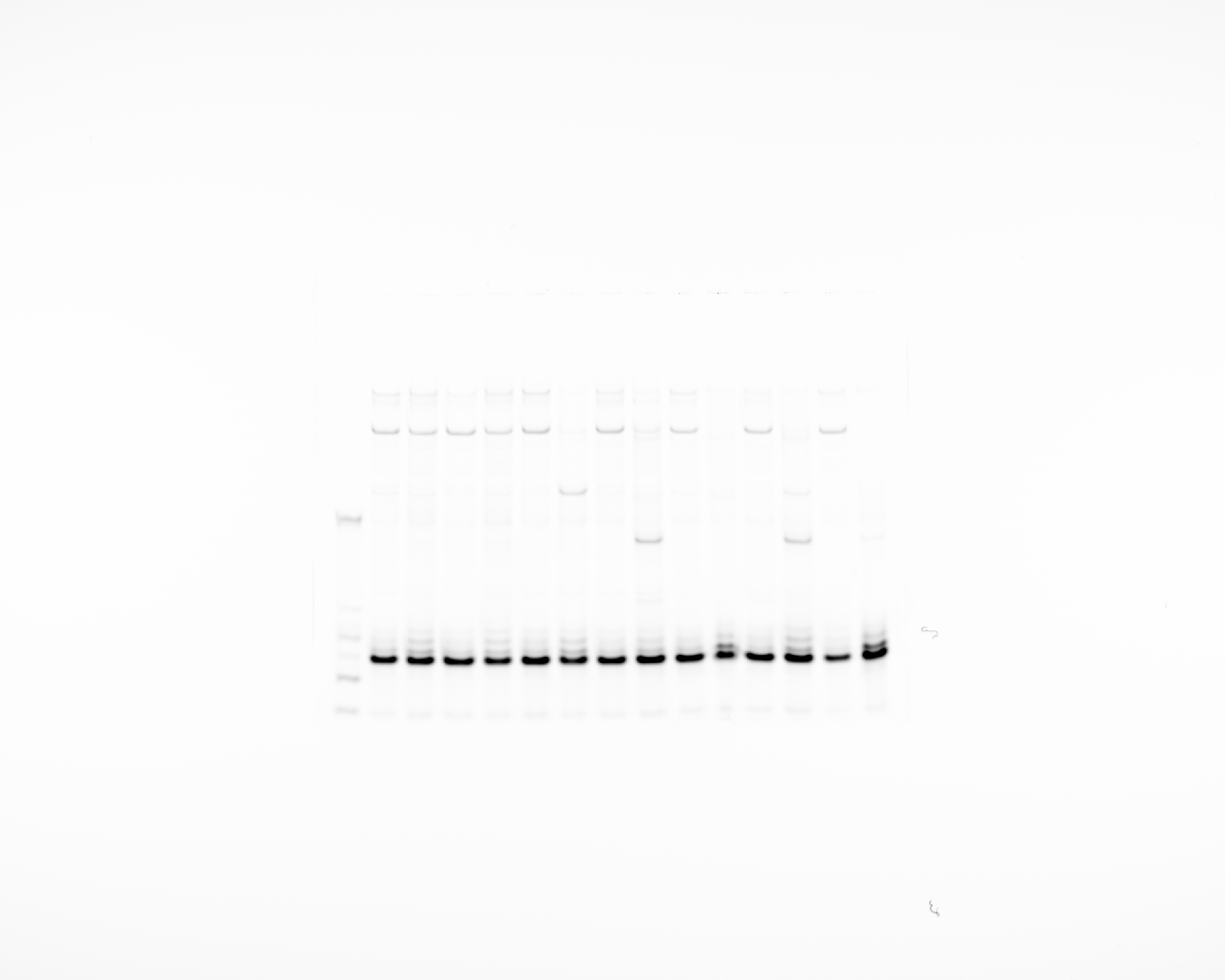


EMBOJ-2025-120166-T_SourceDataForAppendixFigureS11_Ser_top


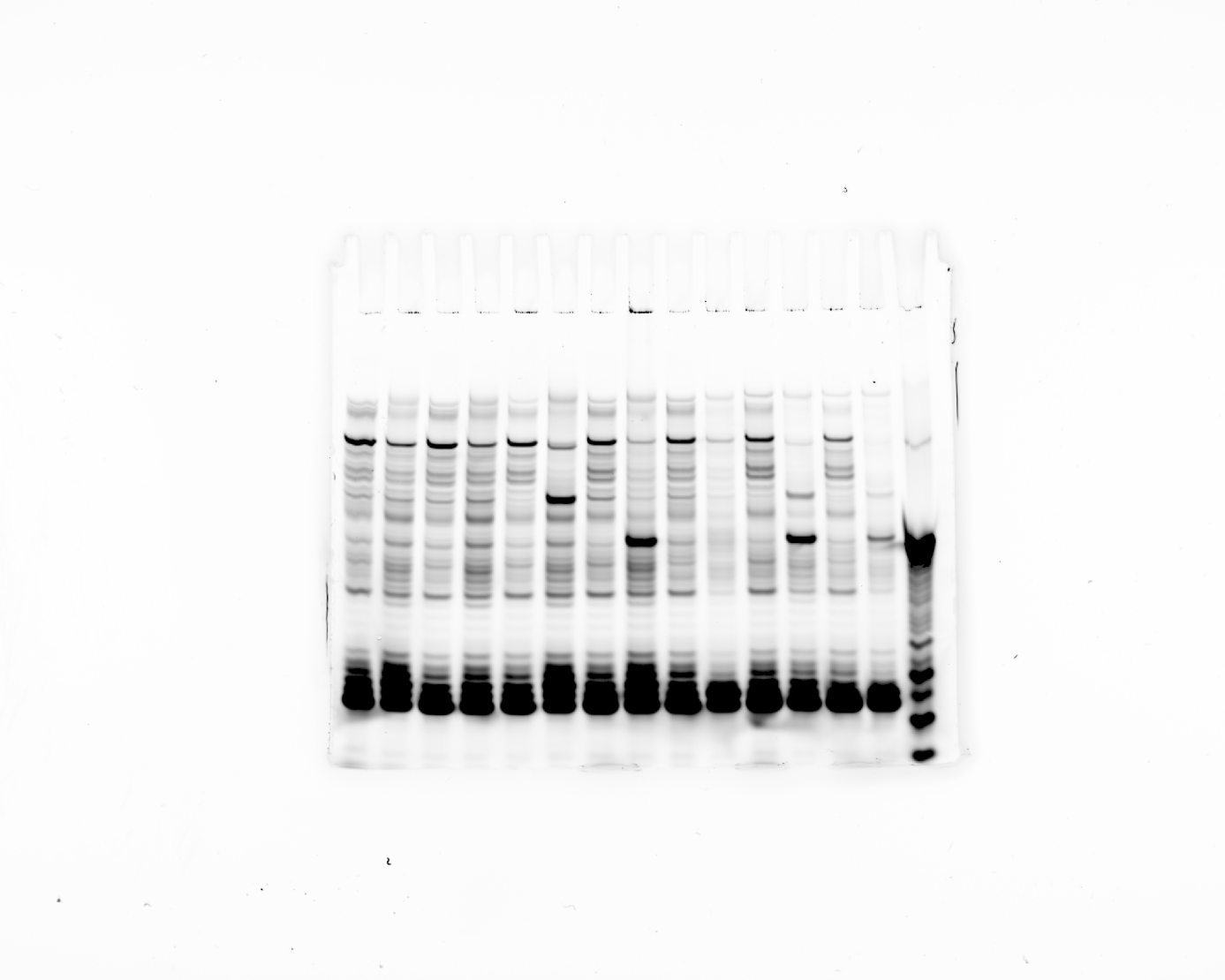


EMBOJ-2025-120166-T_SourceDataForAppendixFigureS11_Ser_bottom


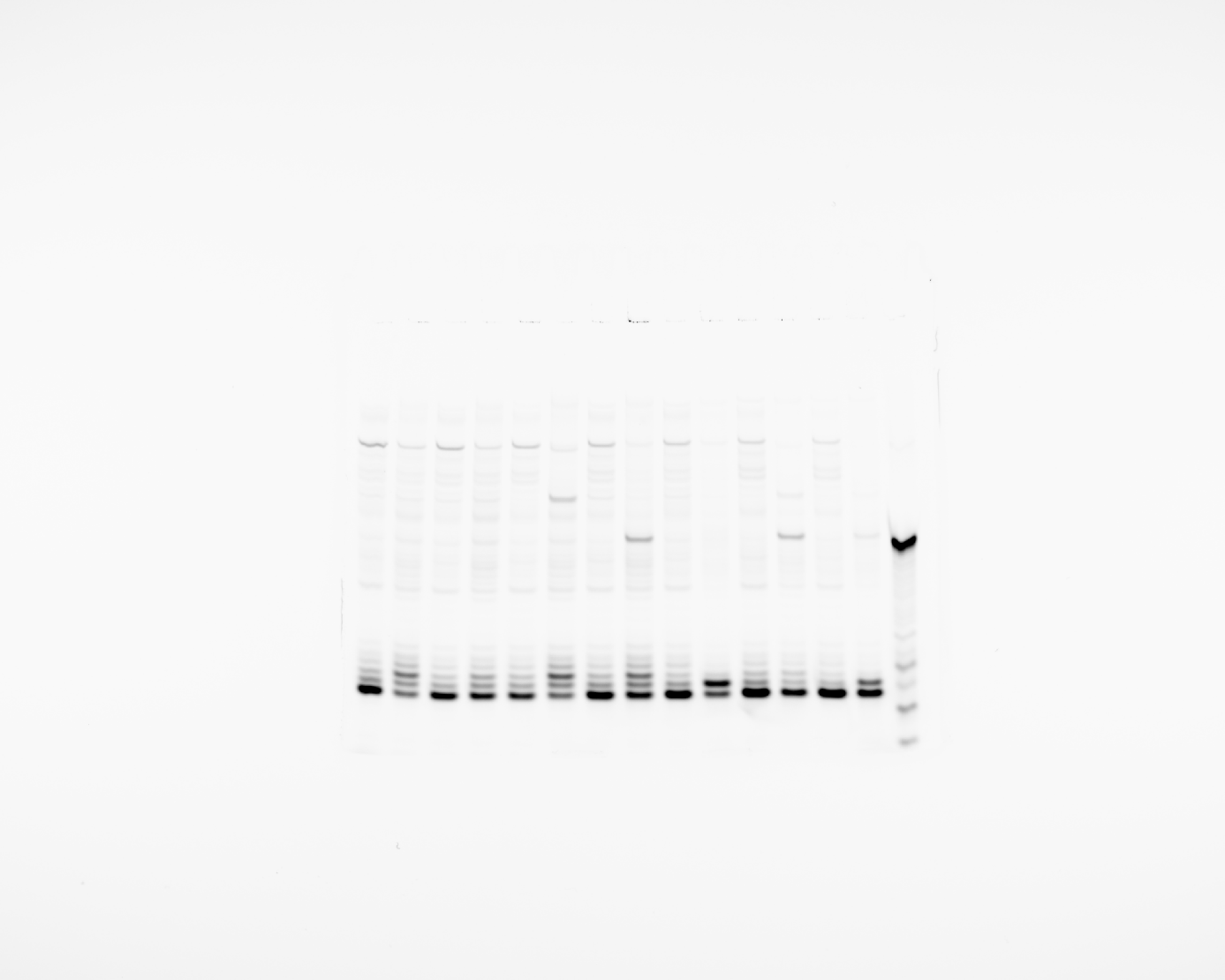


EMBOJ-2025-120166-T_SourceDataForAppendixFigureS11_Arg_top


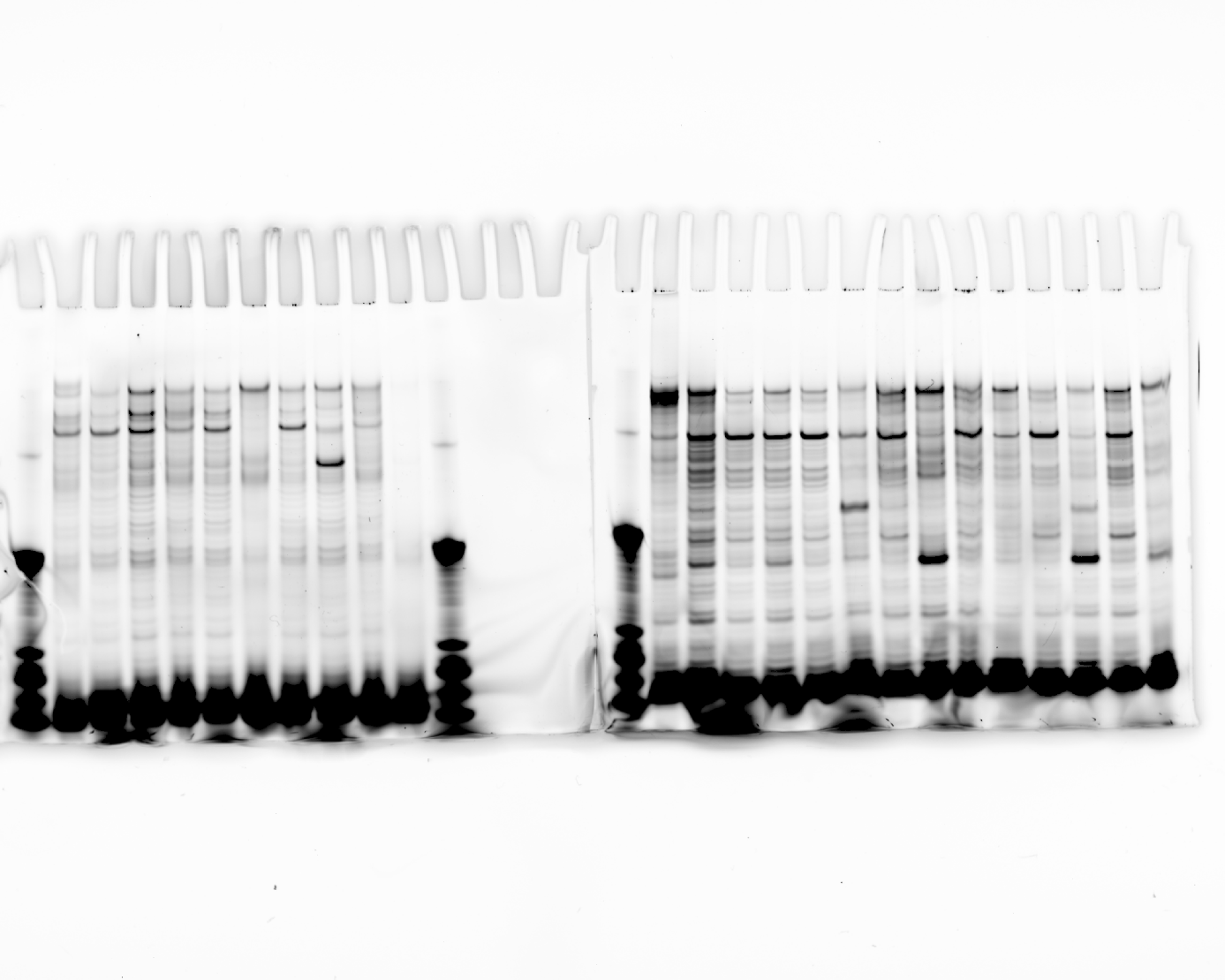


EMBOJ-2025-120166-T_SourceDataForAppendixFigureS11_Arg_bottom


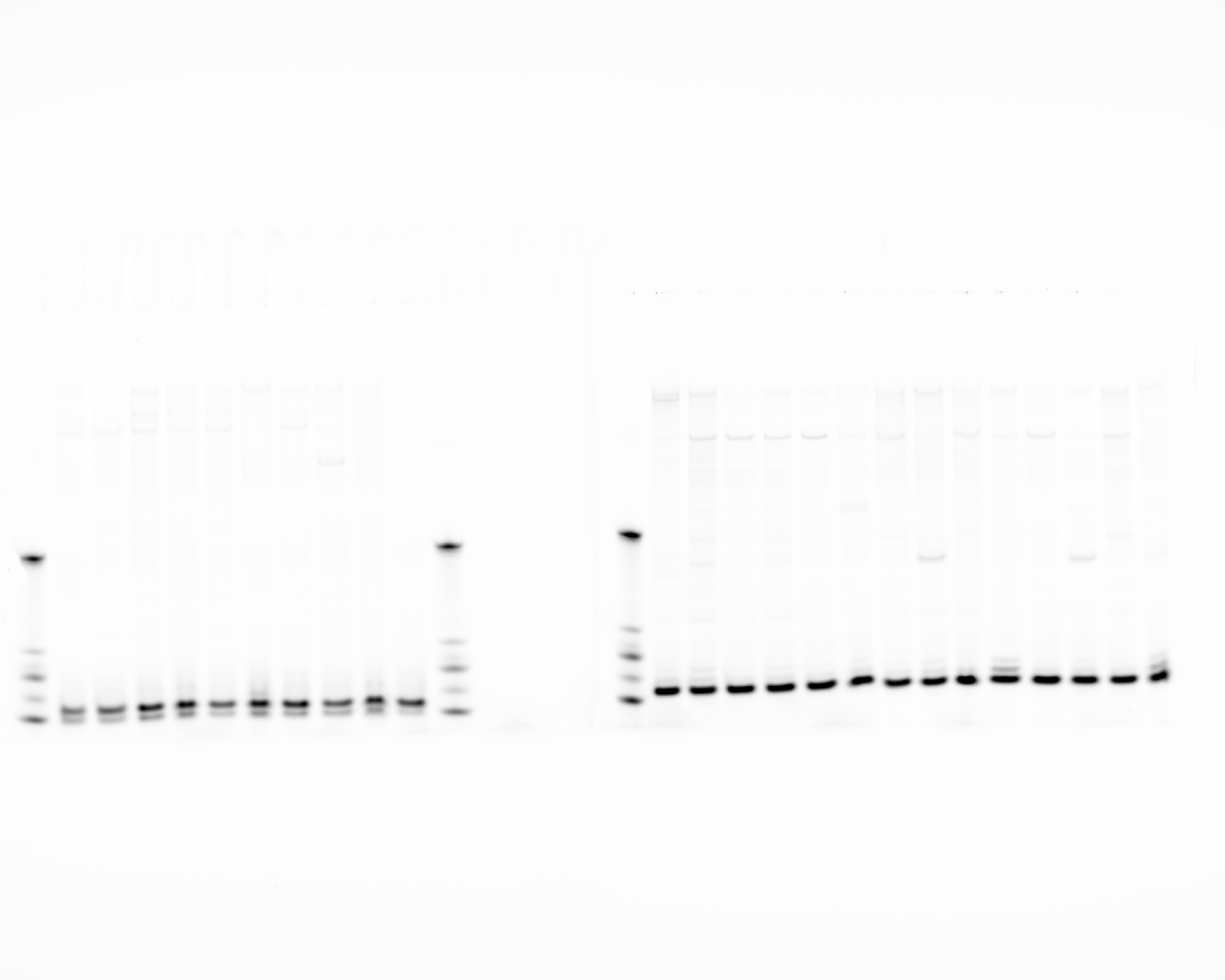

Supplement: Supplementary file 11 — EV and Appendix Figure Source Data [file 44318_2025_443_MOESM11_ESM.zip › EMBOJ-2025-120166-T-SourceData_uncroppedgels_AppendixFigureS11.docx]
