# Supplementary material for: Determining the effects of pseudouridine incorporation on human tRNAs
Source: EMBO J. 2025 Apr 29;44(13):3553–85. doi: 10.1038/s44318-025-00443-y (PMC12217144; doi:10.1038/s44318-025-00443-y)
Supplement: Supplementary file 12 — Expanded View Figures [file 44318_2025_443_MOESM12_ESM.pdf]

Expanded View Figures

Figure EV1. Molecular simulations of tRNA unfolding.

(**A**) NanoDLS analysis of $tRNA^{Gln}_{UUG}$ size changes in the thermal gradient. 2.28 nm rH corresponds to the simulated start of the unfolding process, 2.65 nm rH corresponds to the experimentally calculated inflection point, 3.82 nm rH corresponds to the average value of simulated fully unfolded $tRNA^{Gln}_{UUG}$. (**B**) Conformational space sampled by SimRNA at the different values of the T (temperature) parameter, from very “cold” (0.6) sampling of near-native structures to very “hot” (1.4) sampling of unfolded conformations (left). Relationship between the deviation of the progressively unfolded RNA 3D conformation from the folded structure and the rH value (right). (**C**) Selected models representing each of tRNA unfolding states. Light blue indicates the D-arm, yellow indicate T-arm. (**D**) Calculated hydrodynamic radii of simulated different unfolding states of the $tRNA^{Gln}_{UUG}$ (top). Summary table of mean hydrodynamic radii calculated from the simulated unfolding states of $tRNA^{Gln}_{UUG}$ and their recalibrated values (bottom). (**E**) A heatmap representing the frequency of base-pairing retention in SimRNA simulations at the T parameter ranging from 0.6 to 1.4. Source data are available online for this figure.

© The Author(s)

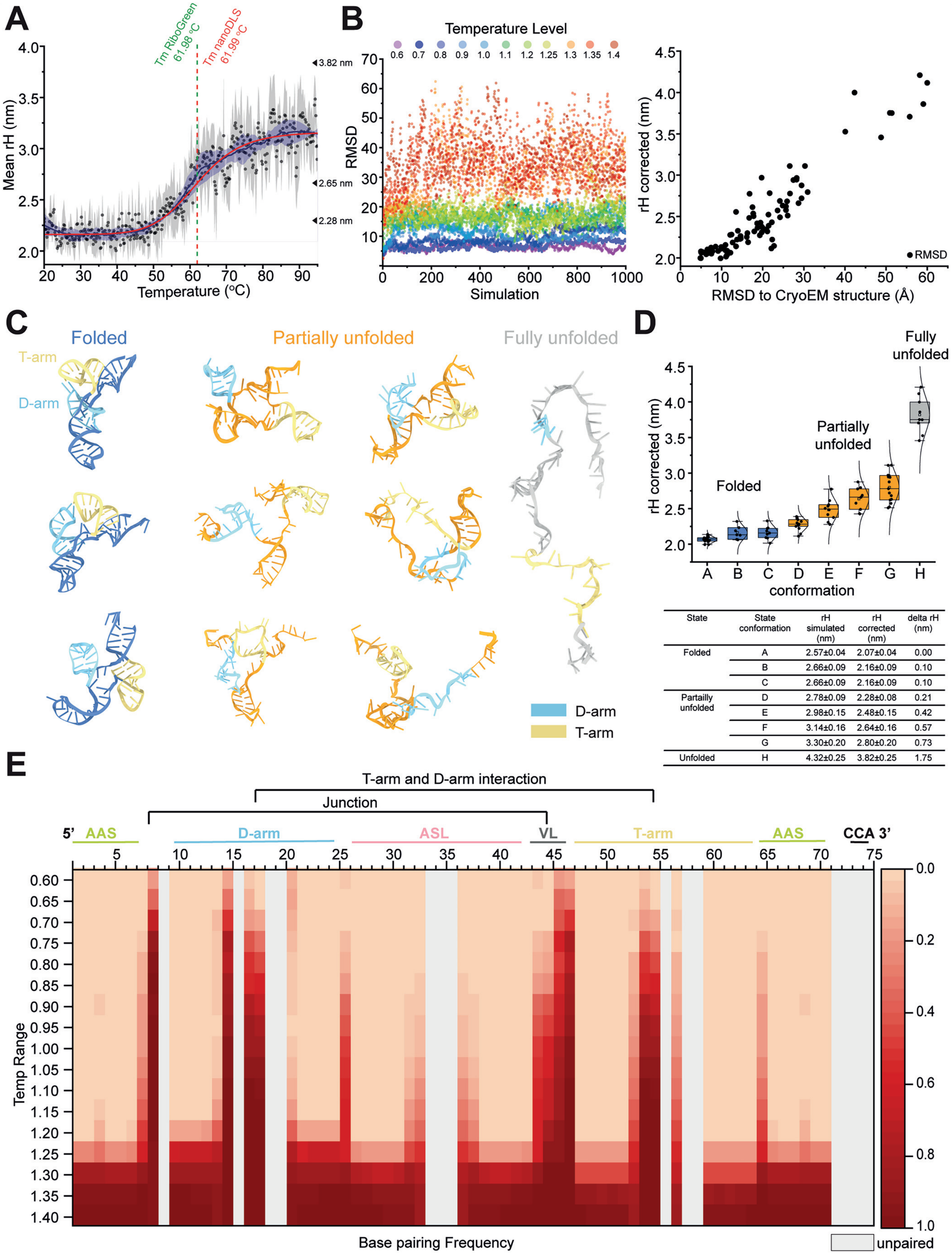

State	State conformation	rH simulated (nm)	rH corrected (nm)	delta rH (nm)
Folded	A	2.57±0.04	2.07±0.04	0.00
	B	2.66±0.09	2.16±0.09	0.10
	C	2.66±0.09	2.16±0.09	0.10
Partailly unfolded	D	2.78±0.09	2.28±0.08	0.21
	E	2.98±0.15	2.48±0.15	0.42
	F	3.14±0.16	2.64±0.16	0.57
	G	3.30±0.20	2.80±0.20	0.73
Unfolded	H	4.32±0.25	3.82±0.25	1.75

© The Author(s)

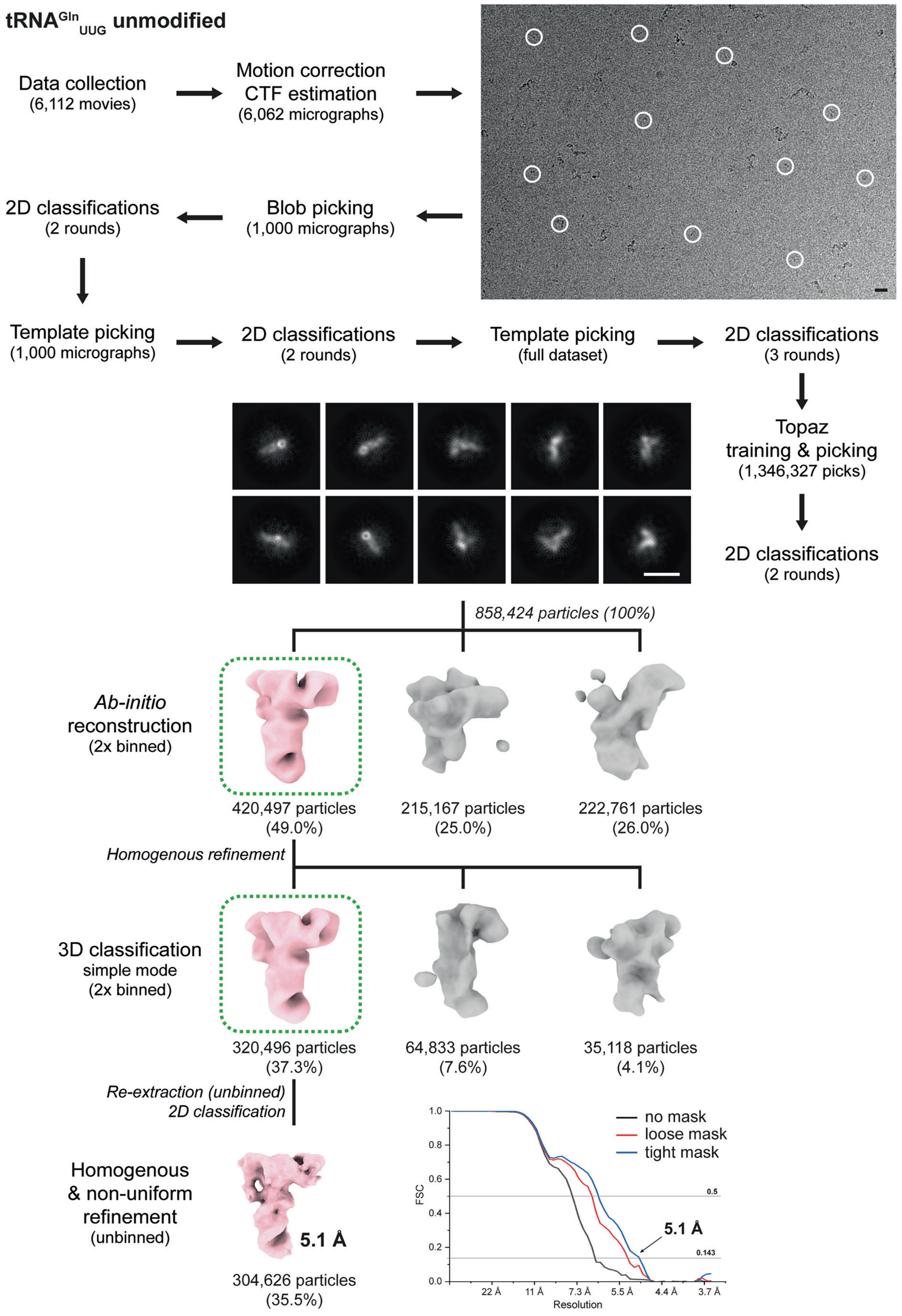
tRNA^Gln_UUG unmodified
Data collection
(6,112 movies)
Motion correction
CTF estimation
(6,062 micrographs)
2D classifications
(2 rounds)
Blob picking
(1,000 micrographs)
Template picking
(1,000 micrographs)
2D classifications
(2 rounds)
Template picking
(full dataset)
2D classifications
(3 rounds)
Topaz
training & picking
(1,346,327 picks)
2D classifications
(2 rounds)
858,424 particles (100%)
Ab-initio
reconstruction
(2x binned)
420,497 particles
(49.0%)
215,167 particles
(25.0%)
222,761 particles
(26.0%)
Homogenous refinement
3D classification
simple mode
(2x binned)
320,496 particles
(37.3%)
64,833 particles
(7.6%)
35,118 particles
(4.1%)
Re-extraction (unbinned)
2D classification
Homogenous
& non-uniform
refinement
(unbinned)
5.1 Å
304,626 particles
(35.5%)
no mask
loose mask
tight mask
FSC
Resolution
22 Å
11 Å
7.3 Å
5.5 Å
4.4 Å
3.7 Å
0.5
0.143

 © The Author(s)

◀ **Figure EV2. Cryo-EM reconstruction pipeline for unmodified $tRNA^{Gln}_{UUG}$.**

A representative micrograph is shown (top right) together with indicated positions of the finally selected particles (white circles); scalebar = 100 Å. Representative 2D classes, ab initio classes and further steps of 3D refinement are shown in the bottom part of the figure. Absolute numbers and percentages of particles are listed and the Fourier Shell Correlation blot (FSC) blot of the final reconstruction, highlighting the nominal resolution at FSC = 0.143.

© The Author(s)

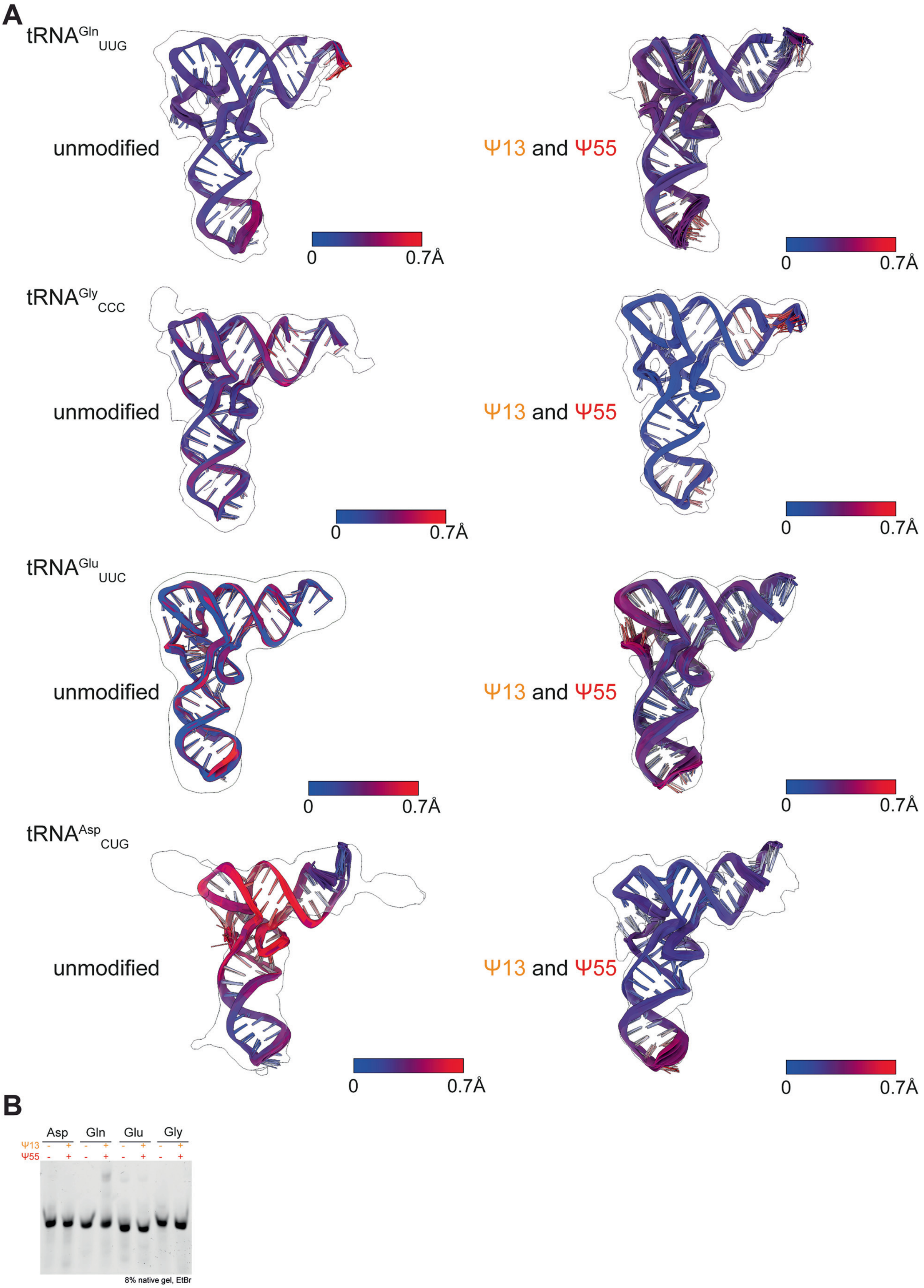
A
tRNAGln UUG
unmodified
Ψ13 and Ψ55
0
0.7Å
tRNAGly CCC
unmodified
Ψ13 and Ψ55
tRNAGlu UUC
unmodified
Ψ13 and Ψ55
tRNAAsp CUG
unmodified
Ψ13 and Ψ55
B
Asp Gln Glu Gly
Ψ13
Ψ55
8% native gel, EtBr

 © The Author(s)

Figure EV3. Comparison of the obtained models within each ensemble.

(**A**) From top to bottom. Cryo-EM reconstructions of unmodified (left) and Ψ_{13}- and Ψ_{55}-modified (right) human RNA^{Gln}_{UUG}, $tRNA^{Gly}_{CCC}$, $tRNA^{Glu}_{UUC}$ and $tRNA^{Asp}_{GUC}$ with an ensemble of 10 atomic models fitted into the density colored by RMSD between each atom. (**B**) A native gel showing the mobility of unmodified and Ψ_{13}- and Ψ_{55}-modified tRNAs.

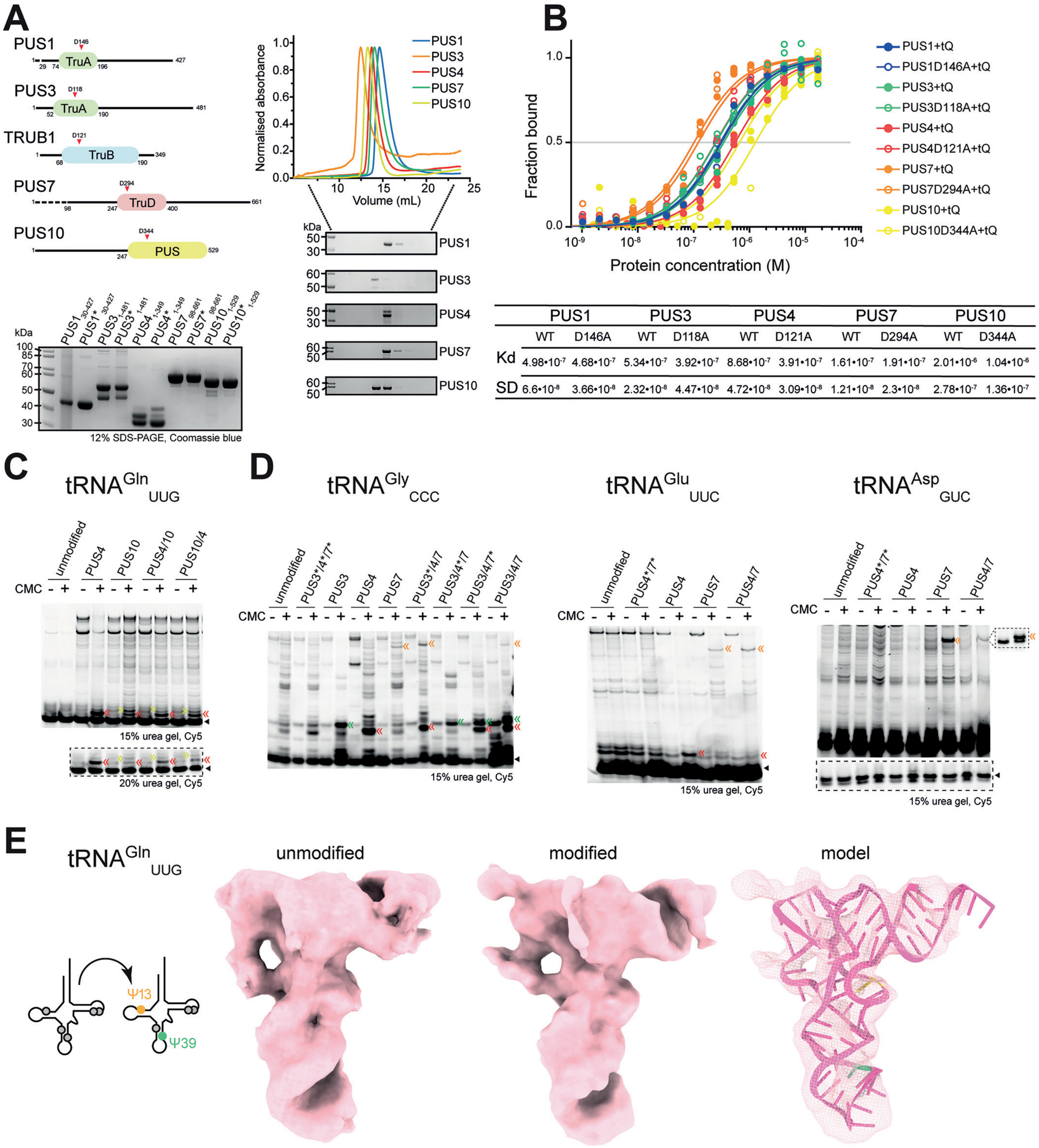

	PUS1		PUS3		PUS4		PUS7		PUS10	
	WT	D146A	WT	D118A	WT	D121A	WT	D294A	WT	D344A
Kd	$4.98 \cdot 10^{-7}$	$4.68 \cdot 10^{-7}$	$5.34 \cdot 10^{-7}$	$3.92 \cdot 10^{-7}$	$8.68 \cdot 10^{-7}$	$3.91 \cdot 10^{-7}$	$1.61 \cdot 10^{-7}$	$1.91 \cdot 10^{-7}$	$2.01 \cdot 10^{-6}$	$1.04 \cdot 10^{-6}$
SD	$6.6 \cdot 10^{-8}$	$3.66 \cdot 10^{-8}$	$2.32 \cdot 10^{-8}$	$4.47 \cdot 10^{-8}$	$4.72 \cdot 10^{-8}$	$3.09 \cdot 10^{-8}$	$1.21 \cdot 10^{-8}$	$2.3 \cdot 10^{-8}$	$2.78 \cdot 10^{-7}$	$1.36 \cdot 10^{-7}$

 © The Author(s)

Figure EV4. In vitro pseudouridinylation of human tRNAs.

(**A**) Cartoon presentation of domains of each PUS (upper-left). The length is labeled, and domain is highlighted where the catalytic residue is indicated by a triangle. The truncated regions of PUS1 and PUS7 are shown as dashed lines. An SDS-PAGE gel of recombinant *Hs*PUS enzymes (lower-left). Catalytic mutants marked with stars. SEC profiles of purified human PUS enzymes (upper-right) and the collected fraction for each purification were resolved in SDS-PAGE gels (lower-right). (**B**) MST analyses of PUS, including wild type and inactive form (DA), binding to $tRNA^{Gln}_{UUG}$. The calculated Kds of wild-type PUS and inactive forms (DA), binding to $tRNA^{Gln}_{UUG}$. (**C**, **D**) Detection of PUS-dependent Ψ formation on $tRNA^{Gln}_{UUG}$, $tRNA^{Gly}_{CCC}$, $tRNA^{Glu}_{UUC}$ and $tRNA^{Asp}_{GUC}$. The reverse-transcribed cDNA products were resolved in a 15% or 18% urea gel and the CMC-Ψ mediated short cDNAs are indicated by double-arrows (Ψ_{13} orange, $\Psi_{27/28}$ blue, Ψ_{39} green, Ψ_{54} yellow Ψ_{55} red). Each tRNA primer (labeled with Cy5) is indicated by a triangle. In the case of $tRNA^{Asp}_{GUC}$, the signal for primer is obtained from a short exposure shown in the dash lined box while the Ψ_{13}-dependent cDNA is obtained using a site-specific primer (shown in a dash lined box on the side). (**E**) Cryo-EM reconstructions of unmodified and Ψ_{13}- and Ψ_{39}-modified $tRNA^{Gln}_{UUG}$. The modified sites are highlighted by color code in the 2D cartoon and the model (Ψ_{13} orange and Ψ_{39} green). Source data are available online for this figure.

© The Author(s)

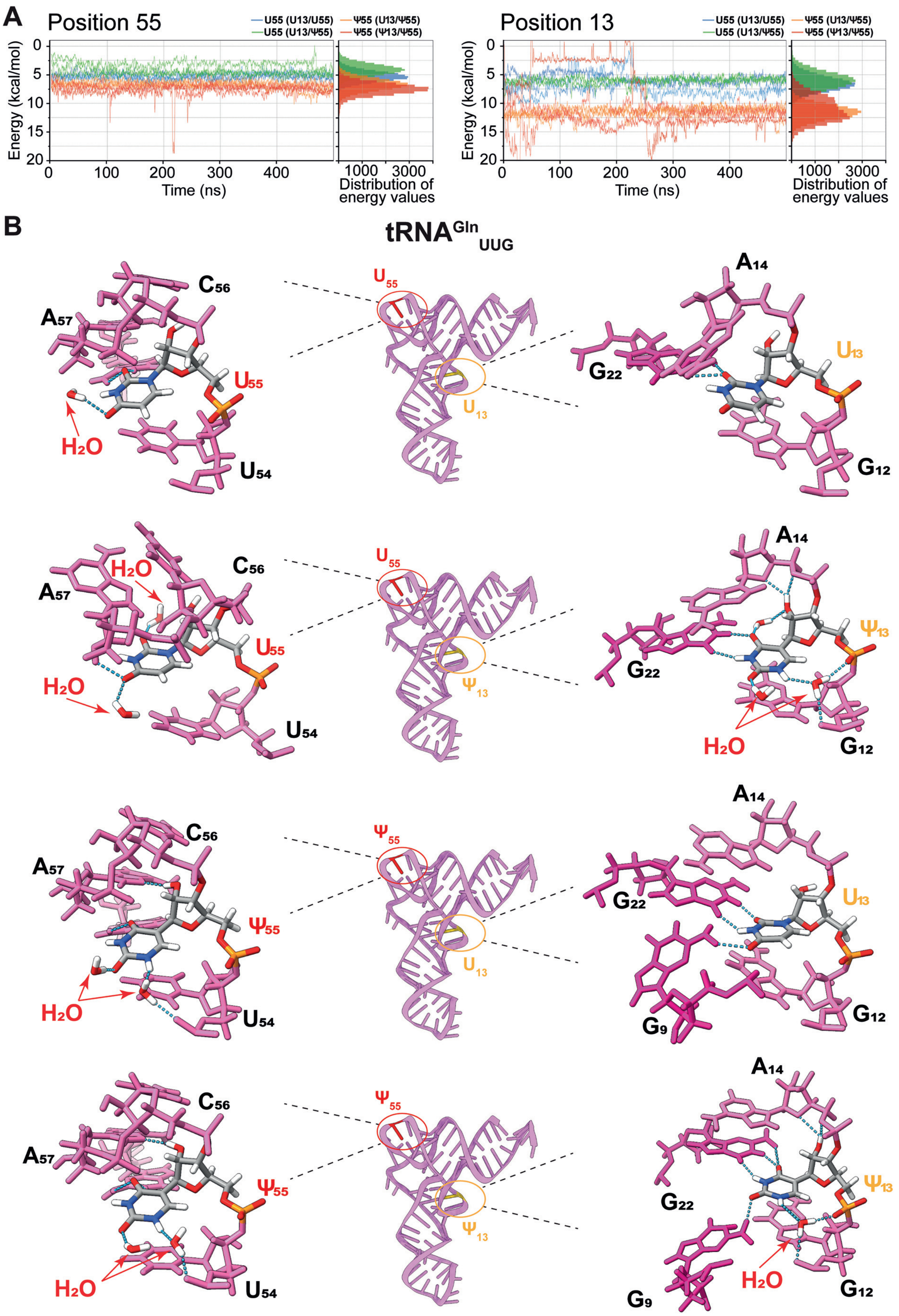
A
Position 55
U55 (U13/U55)
U55 (U13/Ψ55)
Ψ55 (U13/Ψ55)
Ψ55 (Ψ13/Ψ55)
Energy (kcal/mol)
Time (ns)
Distribution of energy values
Position 13
B
tRNAGln UUG
C56
A57
U55
H2O
U54
A14
G22
U13
G12
Ψ13
Ψ55
G9

 © The Author(s)

Figure EV5. MD Simulations of tRNA with and without Ψ_{13} and Ψ_{55}.

(**A**) Distribution of interaction energies between uridine (U) and pseudouridine (Ψ) with neighboring residues throughout the simulation and a histogram of the energy distribution for U_{55}/Ψ_{55} (left) and U_{13}/Ψ_{13} (right). The graphs emphasize the varying stability and dynamics of interactions across different simulation variants. Notably, variants containing Ψ (depicted in orange and red) exhibit greater stability compared to those with U, represented in green and blue. (**B**) Snapshots illustrating the local environments at positions 55 (left) and 13 (right) for all simulation variants. In the simulations where position 13 is occupied by Ψ (specifically in Ψ_{13}/U_{55} and Ψ_{13}/Ψ_{55}), a water molecule (red arrow) forms a triad of hydrogen bonds involving the imino hydrogen of Ψ_{13} and the phosphate groups of both Ψ_{13} and G_{12}. At position 55, when Ψ_{55} is present (in U_{13}/Ψ_{55} and Ψ_{13}/Ψ_{55}), a water molecule (red arrow) bridges between the imino hydrogen of Ψ_{55} and the OP of U_{54}. Notably, these specific interactions are absent when U occupies both the position 13 and 55.

© The Author(s)
